# Reserve Flux Capacity in the Pentose Phosphate Pathway by NADPH Binding Is Conserved across Kingdoms

**DOI:** 10.1016/j.isci.2019.08.047

**Published:** 2019-08-29

**Authors:** Dimitris Christodoulou, Andreas Kuehne, Alexandra Estermann, Tobias Fuhrer, Paul Lang, Uwe Sauer

**Affiliations:** 1 Institute of Molecular Systems Biology, ETH Zurich, Zurich, Switzerland; 2 Systems Biology Graduate School, Zurich 8057, Switzerland

**Keywords:** Biological Sciences, Metabolism, Systems Biology, Metabolic Flux Analysis, Metabolomics, Computational Biology, Bioinformatics

## Abstract

All organisms evolved defense mechanisms to counteract oxidative stress and buildup of reactive oxygen species (ROS). To test whether a potentially conserved mechanism exists for the rapid response, we investigated immediate metabolic dynamics of *Escherichia coli*, yeast, and human dermal fibroblasts to oxidative stress that we found to be conserved between species. To elucidate the regulatory mechanisms that implement this metabolic response, we developed mechanistic kinetic models for each organism's central metabolism and systematically tested activation and inactivation of each irreversible reaction by each metabolite. This ensemble modeling predicts *in vivo* relevant metabolite-enzyme interactions based on their ability to quantitatively describe metabolite dynamics. All three species appear to inhibit their oxidative pentose phosphate pathway during normal growth by the redox cofactor NADPH and relieve this inhibition to increase the pathway flux for detoxification of ROS during stress, with the sole exception of yeast when exposed to high levels of stress.

## Highlights

• Characterization of immediate metabolic response to oxidative stress
• The metabolic response in glycolysis and PP pathway depends on stress severity
• Identification of NADPH feedback inhibition on G6PDH as key regulatory interaction
• The identified oxidative stress regulatory interaction is conserved across kingdoms

## Graphical Abstract

**Figure:**
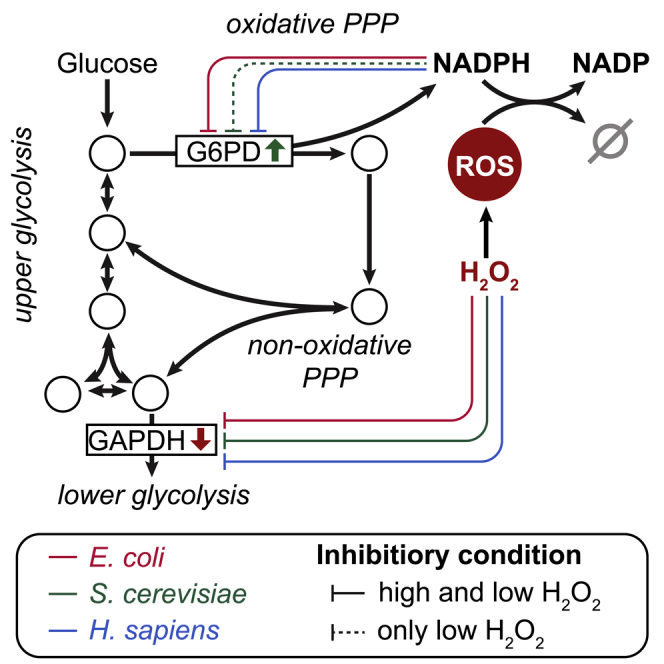

## Introduction

The primeval accumulation of oxygen in the atmosphere was arguably one of the most dramatic changes for life on earth. Although it enabled higher respiratory energy yields due to the high redox potential of oxygen (Raymond and Segrè, 2006), its reactive nature challenges all organisms through reactive oxygen species (ROS), such as hydrogen peroxide (H_2_O_2_), that occur as by-products of aerobic respiration. ROS-dependent oxidation of many cellular constituents such as DNA, proteins, and lipids (Mishra and Imlay, 2012, Imlay, 2013) constitutes a severe threat to cell survival and contributes to a number of human disorders such as cardiovascular diseases, cancer, and aging (Harman, 1981, Alexander, 1995, Waris and Ahsan, 2006, Liou and Storz, 2010).

Long-term transcriptional responses that scavenge ROS appear to be conserved across species (Ralser et al., 2007, Ray et al., 2012, Vatansever et al., 2013, Dan Dunn et al., 2015). In microorganisms, such as *Escherichia coli* and *Saccharomyces cerevisiae*, the coordinated transcriptional response includes the up-regulation of the ROS scavenging superoxide dismutase, catalases, and glutathione/glutaredoxin systems (Godon et al., 1998, Zeller et al., 2007). Similarly, mammalian cells employ long-term anti-oxidative responses that entail ROS detoxification (Morgan and Liu, 2011, Gorrini et al., 2013, Ma, 2013) and, depending on the severity of stress, initiate either pro-survival gene expression programs that support NADPH production, ROS clearance, and DNA repair or cell death programs (Martindale and Holbrook, 2002, Morgan and Liu, 2011, Gorrini et al., 2013, Zhang et al., 2016).

Until transcriptionally regulated defense mechanisms become operational (Chechik et al., 2008), cell survival depends on the basal expression of the above-mentioned enzymes and non-enzymatic antioxidants such as reduced glutathione to scavenge some ROS (Fang et al., 2002, Kohen and Nyska, 2002, Finkel, 2003, Stincone et al., 2015). Increasing evidence points to glutathione peroxidase as one of the key short-term survival mechanisms (Doroshow, 1995, Inoue et al., 1999, Mytilineou et al., 2002, Miyamoto et al., 2003, Ralser et al., 2007, Kuehne et al., 2015, Christodoulou et al., 2018). Glutathione peroxidase-dependent reduction of ROS requires a continuous supply of NADPH for regeneration (Imlay, 2008, Aon et al., 2012). Upon sudden oxidative stress, the glutathione-based detoxification of ROS and the concomitant oxidation of NADPH drastically decreases the NADPH pool that must be rapidly replenished. The major replenishing reactions are catalyzed by glucose 6-phosphate (G6P) dehydrogenase and phosphogluconate (6PG) dehydrogenase in the oxidative branch of the pentose phosphate (PP) pathway of bacteria, *S. cerevisiae*, and most mammalian cells (Fuhrer and Sauer, 2009, Stincone et al., 2015). Upon oxidative stress, all cells increase the reduction rate of NADP^+^ to NADPH mainly by rerouting their glycolytic flux into the PP pathway (Ralser et al., 2007, Rui et al., 2010, Anastasiou et al., 2011, Kuehne et al., 2015, Christodoulou et al., 2018).

For *E. coli* we recently demonstrated this rapid flux rerouting to be achieved primarily by the relief of G6P dehydrogenase inhibition from NADPH that liberates the reserve flux capacity of the PP pathway (Christodoulou et al., 2018). Together with the blockage of lower glycolysis caused by direct oxidation of key enzymes (i.e., glyceraldehyde 3-phosphate dehydrogenase [GAP dehydrogenase] or pyruvate kinase M2 in mammalian cells) (Colussi et al., 2000, Ralser et al., 2007, Ralser et al., 2009, Anastasiou et al., 2011), this mechanism is sufficient to explain the rapid metabolic adaptation in *E. coli* (Christodoulou et al., 2018). This view is consistent with recent findings of an NADPH-dependent activation of oxidative PP pathway fluxes upon oxidative stress in human dermal fibroblasts (Kuehne et al., 2015). For the lower eukaryote *S. cerevisiae*, the short-term oxidative stress response has been suggested to depend primarily on blockage of lower glycolysis (Ralser et al., 2007, Ralser et al., 2009). To elucidate whether the reserve PP pathway flux capacity and the mechanisms that liberate it for enhanced oxidative stress survival are conserved across kingdoms of life, we characterized the immediate metabolic response of *E. coli*, *S. cerevisiae*, and human dermal fibroblasts to low and high oxidative stress. Multivariate and timing analysis revealed a conserved metabolome between species, and mechanistic modeling with ensembles of thousands of models of glycolysis and PP pathway, with different combinations of regulatory mechanisms for each of the species, revealed that alleviation of NADPH inhibition of G6P dehydrogenase, is a conserved and highly important mechanism for the rerouting of flux in every cell type and stress intensity, with the only exception of *S. cerevisiae* when exposed to high levels of stress.

## Results

### The Immediate Metabolic Response upon Exposure to Oxidative Stress

**Figure 1:**
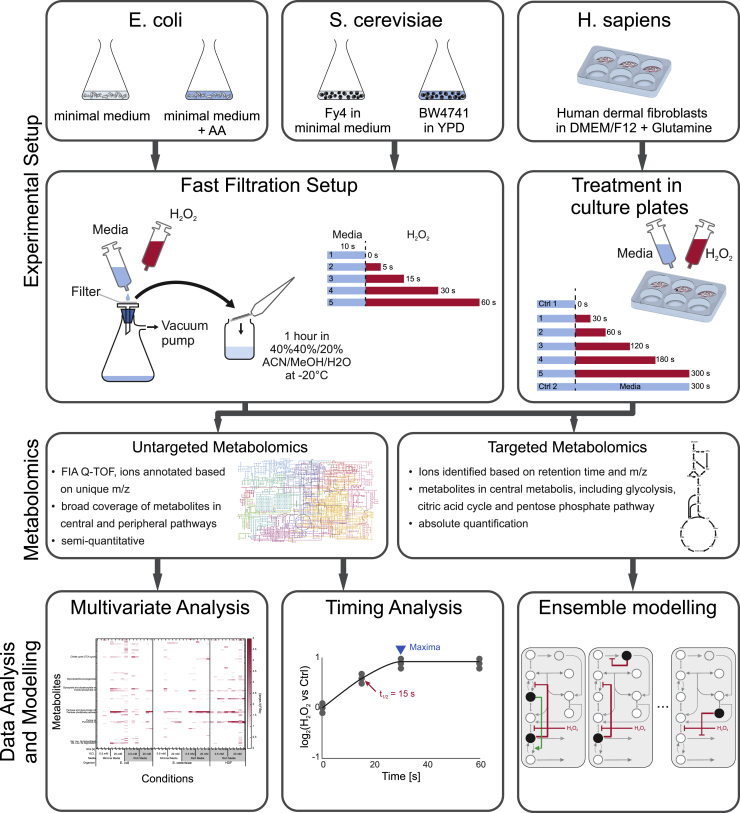
Experimental— – Computational Workflow Triplicate cultures of *E. coli* and *S. cerevisiae* were grown in rich and minimal medium and human dermal fibroblasts in rich medium. Mid exponential growth phase cultures of microbes were transferred to a filter and for 10 s perfused with the cultivation medium and then with the same medium but with either a low (0.5 mM) or high (20 mM) dose of H_2_O_2_. The mammalian stress experiments were performed in liquid culture through addition of H_2_O_2_ dosage. Culture aliquots were immediately transferred into approximately −20°C cold quenching/extraction liquid and prepared for mass spectrometric analysis of the intracellular metabolome. Using the data from the untargeted metabolomics measurements, we performed multivariate analysis and timing analysis. To systematically map all metabolite-enzyme interactions and their functional relevance, we developed kinetic models of glycolysis and the PP pathway for all species and conditions. Ensembles of models with different putative regulatory interactions were then tested for their ability to capture the dynamics of eight metabolites in central metabolism.

To compare the immediate metabolic response to oxidative stress between *E. coli*, *S. cerevisiae*, and human dermal fibroblasts, we challenged exponentially growing cultures with low (0.5 mM) and high (20 mM) levels of H_2_O_2_. Before the stress, *E. coli* and *S. cerevisiae* were grown in minimal and rich medium (Figure 1) to assess the influence of growth rate and condition. Given previous knowledge on the dynamics of the oxidative stress responses (Ralser et al., 2009) (Kuehne et al., 2015) (Christodoulou et al., 2018), dynamic metabolome profiles were determined in triplicate experiments during 1 min for *E. coli* and *S. cerevisiae* and 5 min for human dermal fibroblast post stress. The employed non-targeted mass spectrometry method (Fuhrer et al., 2011) allowed us to annotate 230 measured ions to 467 metabolites listed in the KEGG metabolite database (Kanehisa and Goto, 2000, Kanehisa et al., 2017) based on the mass-to-charge ratio using a strict tolerance of 0.001 amu (Table S1).

Consistent with previous data (Christodoulou et al., 2018, Ralser et al., 2007, Ralser et al., 2009, Kuehne et al., 2015), all three species responded rapidly already to the lower H_2_O_2_ challenge (Figures S1 and S2). Under all tested conditions, we observed an immediate metabolic response at the earliest measured time point (5 s for *E. coli* and *S. cerevisiae* and 30 s for human cells) that steadily progressed over time (Figure S1). Specifically, the ratio of oxidized to reduced glutathione increased after only 5 s (human dermal fibroblasts [HDF]: 30 s) and stabilized after about 10 s (HDF: 60 s), most pronounced upon treatment with 20 mM H_2_O_2_ (Figure S2A). This rapid increase was conserved across almost all species and conditions. Only treatments with 0.5 mM H_2_O_2_ in yeast cultivated in rich media and human cells resulted in a continuous increase of the oxidized to reduced glutathione ratio. Remarkably, the ratio of oxidized to reduced glutathione was higher, in particular for *E. coli* in minimal medium and yeast in rich medium (Figure S2B). This observation indicates that *E. coli* has a lower capacity to cope with high oxidative stress in minimal medium, which could explain the greater increase of the oxidized to reduced glutathione ratio at high stress in rich medium (>10-fold increase) compared with minimal medium (∼2-fold increase).

**Figure 2:**
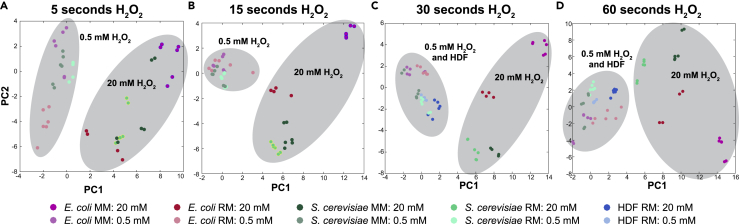
The Immediate Metabolic Response to Oxidative Stress The immediate metabolic response upon exposure to oxidative stress for (A) 5 s, (B) 15 s, (C) 30 s, and (D) 60 s. The axis shows the first two principal components of a principal component analysis of the metabolomics data of cells treated for 30 and 60 s with H_2_O_2_. (A) and (B) are without data for HDF cells since shortest treatment was 30 s.

Pathway enrichment analysis of metabolite changes at each time point (compared with untreated controls) revealed glycolysis, gluconeogenesis, tricarboxylic acid cycle, PP pathway, glyoxylate, amino acid, and purine and pyrimidine metabolism as the first responders (Figure S3). The changes in central and nucleotide metabolism exhibited a high degree of similarity across species and conditions (Figures S4–S9), suggesting a conserved response, consistent with reports on individual species (Colussi et al., 2000, Shenton and Grant, 2003, Murakami et al., 2006, Ralser et al., 2007, Ralser et al., 2009, Anastasiou et al., 2011). Stress intensity had the strongest impact on the metabolic response because the first principal component of the metabolomics data separated the samples based on stress level of H_2_O_2_ at every treatment duration (Figure 2). The pre-stress growth condition was less relevant than cell type because most samples clustered according to cell type, with the exception of the 5 s time point of *E. coli* at high and low stress.

### Timing Analysis Reveals Conservation of the Metabolic Response Dynamics

**Figure 3:**
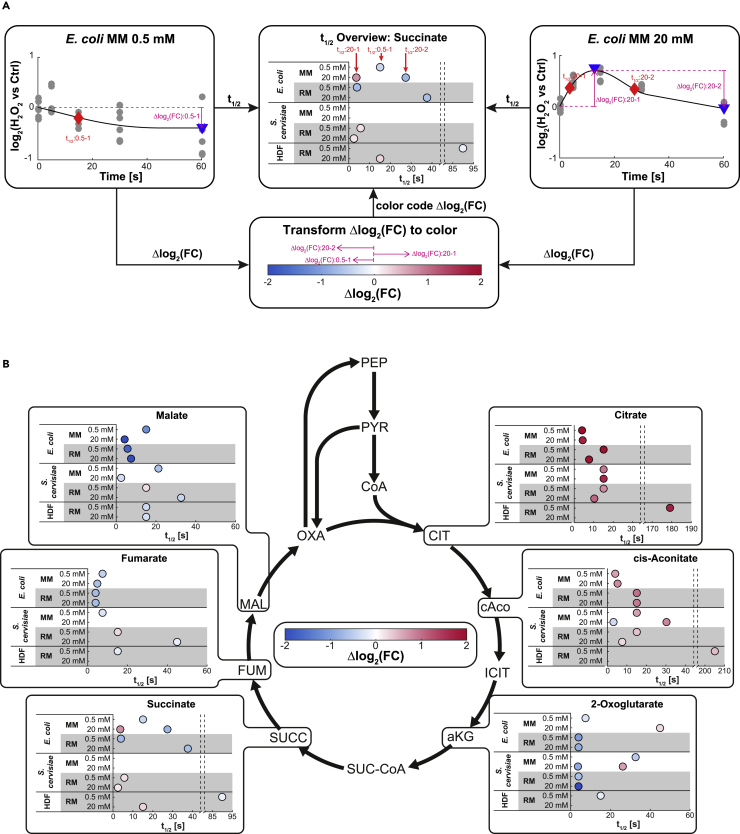
Timing Analysis and Its Results for the Citric Acid Cycle (A) Timing analysis. To determine the timing of the metabolic change upon H_2_O_2_ exposure, multivariate adaptive regression splines were fit to each temporal trace (log2[x vs 0 min H_2_O_2_]) of each metabolite. (B) Timing analysis results for the citric acid cycle. Timing analysis results for all organisms and conditions considered, for the intermediates of the citric acid cycle. The half-time to local maximum t_1/2_ was not determined (1) for spline fits with R2 < 0.2 and (2) if no significant maxima could be identified (i.e., peak prominence of Δlog2(FC) < 0.2). Furthermore, following local maxima with less than 50% change of log_2_(FC) were removed.

To elucidate whether the conserved pathway responses were also similar in terms of their dynamics, we determined the time needed for every measured metabolite to reach half of its maximum fold change (T_1/2_) (Figures 3A and S10–S14). In the tricarboxylic acid cycle, we observed continuous—up to 4-fold—increase of *cis*-aconitate (except for HDF 20 mM) and citrate and a decrease of fumarate and malate in all species, for all stress intensities and in both media (Figures 3B and S6). These results are consistent with the strong reduction of TCA cycle activity due to inhibition of isocitrate dehydrogenase, aconitase, and alpha-ketoglutarate dehydrogenase upon exposure to oxidative stress (Murakami et al., 2006, Sandoval et al., 2011, Tretter and Adam-Vizi, 2000, Tretter and Adam-Vizi, 2005). Succinate did not consistently decrease in all cases, which could be explained by a potential direct conversion of aKG to succinate to neutralize ROS (Liu et al., 2018).

**Figure 4:**
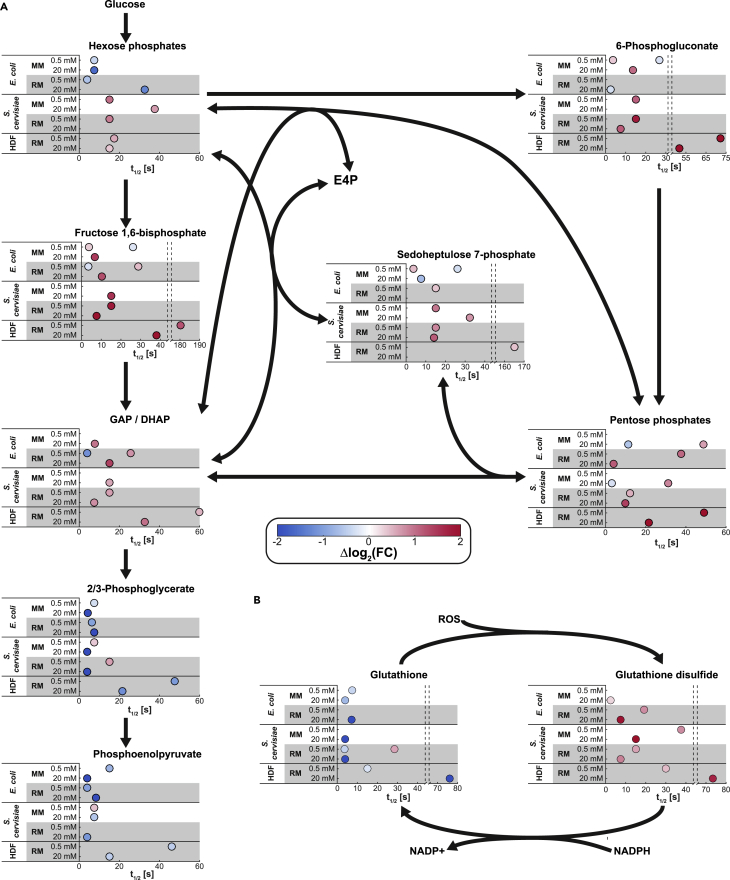
Timing Analysis Results for Glycolysis, the PP Pathway, and the Glutathione System (A) Timing analysis results for all organisms and conditions considered for the intermediates of glycolysis and the PP pathway. (B) Timing analysis results for all organisms and conditions considered for the glutathione regeneration mechanism. The half-time to local maximum t_1/2_ was not determined (1) for spline fits with R2 < 0.2 and (2) if no significant maxima could be identified (i.e., peak prominence of Δlog2(FC) < 0.2). If more than one local maximum is detected (>50% change of log_2_(FC)), a half-time for the change between subsequent local maxima is determined.

Glycolysis dynamics were also consistent across organisms, with fructose 1,6-bisphosphate and GAP/dihydroxyacetone phosphate (DHAP) increasing and metabolites of lower glycolysis such as 2/3-phosphoglycerate and phosphoenolpyruvate (PEP) decreasing (Figures 4 and S5). The response of hexose phosphates was not conserved because *E. coli* exhibited an opposite effect compared with the increase in *S. cerevisiae* and human cells. Finally, we found the PP pathway dynamics to be highly conserved across species and conditions (except *E. coli* in rich media and yeast in minimal media under high stress), with the strongest immediate increase for 6PG in the oxidative branch of the PP pathway. The levels of metabolites in the non-oxidative branch, like sedoheptulose 7-phosphate (S7P) and pentose phosphates, showed a synchronous dynamic increase, except for the high stress in *E. coli*. Interestingly, under low-stress conditions accumulation of PP pathway metabolites is faster or as fast as accumulation of upper glycolytic metabolites (Figures 4 and S15). In contrast, under some high-stress conditions (yeast in both media and *E. coli* in minimal medium), accumulation of glycolytic intermediates precedes accumulation of PP pathway intermediates.

Thus, our analysis demonstrates that the short-term dynamic responses in central metabolism are largely conserved across all tested cell types, where high stress levels induced faster responses across all cell types and the human cell line responded on average five times slower than *E. coli* and *S. cerevisiae* (Figures 3B and 4). However, the detailed mechanisms that are involved in this rerouting and if they are conserved remain unclear.

### Model-Based Identification of a Conserved Mechanism that Enables Rapid Adaptation to Oxidative Stress

How is this conserved metabolic response mechanistically achieved in the different species? In *E. coli* and human dermal fibroblasts, the rapid flux rerouting from glycolysis to the PP pathway under low-stress conditions is controlled by the relief of G6P dehydrogenase inhibition through dropping NADPH levels in combination with the ROS-mediated blockage of lower glycolysis (Christodoulou et al., 2018) (Kuehne et al., 2015). For yeast under high oxidative stress latest studies point toward the latter mechanism (Ralser et al., 2009). Rapidly depleting intermediates of lower glycolysis (i.e., xPG, PEP) in all organisms and conditions demonstrate the blockage of lower glycolysis upon stress, most likely due to oxidation of GAP dehydrogenase (Ralser et al., 2009, Kuehne et al., 2015). Consistent with the *E. coli* model and previous findings in mammalian cells (Kuehne et al., 2015), our timing analysis revealed a generally faster and much stronger increase in the first PP pathway intermediate (6PG) and pentoses than in the glycolytic hexoses and fructose 1,6-bisphosphate (Figures 4 and S5). In three cases we found a synchronous increase in the levels of hexoses or fructose 1,6-bisphosphate compared with 6PG and pentoses, i.e., *S. cerevisiae* stressed with 0.5 mM H_2_O_2_ in minimal or rich medium and mammalian cells stressed with 20 mM H_2_O_2_ (Figures 4 and S5). The only case with a faster response in the hexoses compared with 6PG and pentoses was *S. cerevisiae* grown in minimal medium and stressed with 20 mM H_2_O_2_ (Figures 4 and S5).

Overall, our results are consistent with a direct activation of the oxidative PP pathway rather than a passive flux rerouting as a consequence of the glycolytic block, with the sole exception of *S. cerevisiae* in minimal medium and high stress. It is precisely for this condition that blockage of lower glycolysis was suggested to be largely sufficient to shift NADPH-producing fluxes into the PP pathway of yeast (Ralser et al., 2009). Thus, our results are in good agreement with previous results (Ralser et al., 2009) (Kuehne et al., 2015) (Christodoulou et al., 2018), but our timing analysis does not allow us to conclude whether this blockage alone is sufficient to explain the metabolite dynamics in every organism and condition or whether the hypothesized reserve capacity of flux in G6P dehydrogenase is also needed.

To verify whether both regulatory mechanisms are required for all three organisms and to clarify the discrepancy between the low and high oxidative stress treatment in *S. cerevisiae* (Ralser et al., 2009), we developed kinetic models for each of the three organisms. Kinetics of reversible and irreversible reactions were modeled with mass action and Michaelis-Menten laws, respectively, as described before (Christodoulou et al., 2018). Each model consisted of 12 ordinary differential equations, with 12 metabolites and 24–26 reactions that represent glycolysis, PP pathway, and glutathione detoxification of ROS by the oxidation of NADPH, which represents the perturbation (see also Transparent Methods, Kinetic Model of Glycolysis/Gluconeogenesis and the Pentose Phosphate Pathway for more information). Kinetic enzyme parameters (Table S2) and specific glucose uptake rates were obtained from the literature, where *E. coli* and *S. cerevisiae* are reported to feature similar uptake rates in the range of 1–2 mM/s (Christen and Sauer, 2011, Zampar et al., 2014, Gerosa et al., 2015, Park et al., 2016). The glucose uptake rate for human cells is approximately two orders of magnitude lower, in the range of 0.02–0.2 mM/s (Lemons et al., 2010, Park et al., 2016). To account for the parametric uncertainty, the Michaelis-Menten constants (K_M_) of each enzyme were randomly sampled 2,000 times in a 0.1–10 times range around their literature values, and maximum reaction rates (V_max_) were calculated from flux distributions during steady-state growth on glucose (Link et al., 2013) (see Transparent Methods, Kinetic Model of Glycolysis/Gluconeogenesis and the Pentose Phosphate Pathway for more information). Owing to these broad ranges in parameter sampling, we adequately considered uncertainty in kinetic parameters, uptake rates, and flux distributions in the different organisms.

To evaluate species differences with our kinetic models, we quantified absolute intracellular concentrations of 30 metabolites by a targeted liquid chromatography-tandem mass spectrometry method (Buescher et al., 2010), for the same conditions and time points as before (Table 1). The models amended with only ROS inhibition of lower glycolysis could explain accumulation of upper glycolytic metabolites, such as FBP and GAP/DHAP in particular for the high-stress conditions, but could not capture metabolite dynamics in the PP pathway (Figure S16). To identify additionally relevant, putative metabolite-enzyme regulation, we systematically tested activation and inactivation of every irreversible reaction by each of the 12 metabolites through adding a power law term that affects the maximum reaction rate, as described previously (Christodoulou et al., 2018). For each organism, we thus generated an ensemble of 10,000–12,000 structurally different models, each consisting of the base model with ROS inhibition of GAP dehydrogenase, plus two putative metabolite-enzyme interactions. The approximately 120 million simulations—2,000 simulations per model, organism, and stress level—were performed with an efficient pipeline based on parallel computing principles (see Transparent Methods, Kinetic Model of Glycolysis/Gluconeogenesis and the Pentose Phosphate Pathway for more information, section Parallel ensemble modeling framework), as previously described (Christodoulou et al., 2018).

**Table 1:** Summary of the Different Experimental Conditions

Species	Medium	H_2_O_2_ Concentration (mM)	Time Points Sampled (s)
*E. coli*	Rich medium (M9 + AA + glucose)	0.5	0, 5, 15, 30, 60
		20	0, 5, 15, 30, 60
	Minimal medium (M9 + glucose)	0.5	0, 5, 15, 30, 60
		20	0, 5, 15, 30, 60
*S. cerevisiae*	Rich medium (YPD)	0.5	0, 5, 15, 30, 60
		20	0, 5, 15, 30, 60
	Minimal medium (M9 + glucose)	0.5	0, 5, 15, 30, 60
		20	0, 5, 15, 30, 60
Human dermal fibroblasts (HDF)	Rich medium	0.5	0, 30, 60, 120, 180, 300
		20	

*E. coli* and *S. cerevisiae* were subjected to different environments represented by rich medium and minimal medium. HDF was grown only in rich medium as growth on minimal medium could not be achieved. In addition, each environmental condition was exposed to two different stress levels during the experiment. This corresponds to two different concentrations of H_2_O_2_ (0.5 and 20 mM). In total ten different conditions were obtained and analyzed.

We use this modeling framework primarily as a hypothesis generation tool by asking whether putative regulatory interactions (or combinations thereof) are able to capture the dynamic responses to oxidative stress better than models without regulatory interactions. To identify those interactions that occur most probably *in vivo*, the 2,000 simulated metabolome responses of each model are compared with the experimentally determined ones. Putative interactions occurring frequently in better scoring models are considered to be more likely. With the sole exception of the high stress challenge in *S. cerevisiae*, additional allosteric interactions strongly improved the description of the metabolite dynamics, in particular for upper glycolytic and PP pathway intermediates (see Figure S16 and Tables S4, S5, S6, S7, S8, S9, and S10 for quantitative evaluation of the improvement). Please note that none of the models was actually fitted to the data, hence the predicted responses from 2,000 randomly chosen parameter sets are not expected to fit the data perfectly.

**Figure 5:**
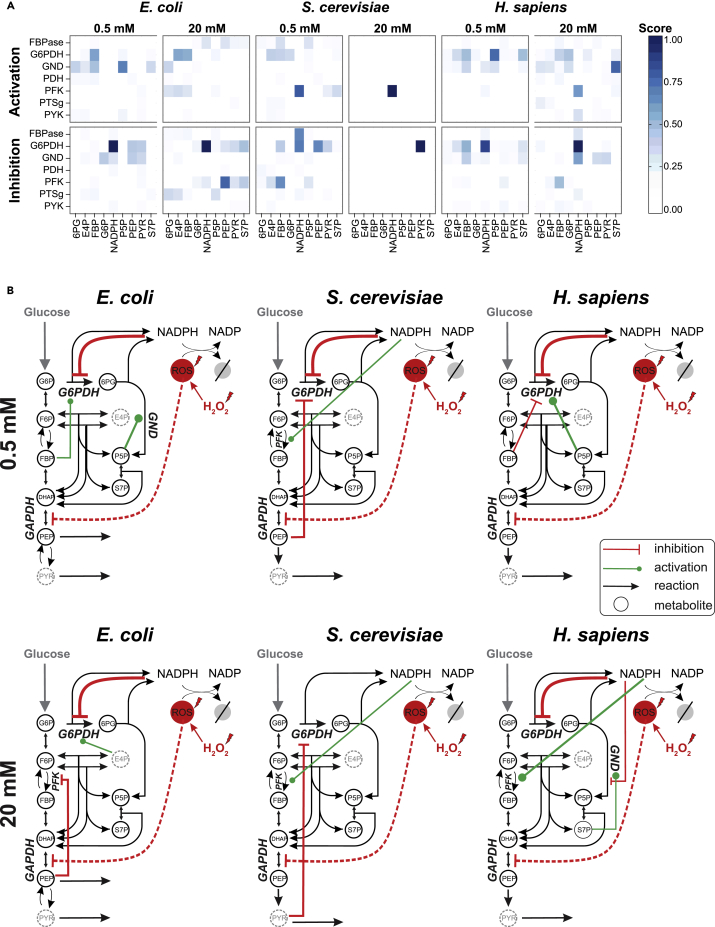
Results from the Model-Based Identification of Mechanisms that Enable Rapid Adaptation and the Conserved Inhibition of G6P Dehydrogenase by NADPH (A) Heatmap depicting the overall rank achieved by different interactions (activating or inhibiting enzymes) in *E. coli, S. cerevisiae*, and *H. sapiens* in different stress levels. For *E. coli* and *S. cerevisiae* the minimal medium condition was used, whereas for *H. sapiens* the only condition available (rich medium). The darker the blue, the higher the rank of the interaction, and therefore its importance. (B) Depiction of the glycolysis and PP pathway circuitry for every organism and stress level in minimal medium we considered. Besides the known inhibition of lower glycolysis by ROS, the best three interactions for each organism, for each stress are depicted on every diagram.

To identify the specific regulatory interactions that improved description of the data we used two measures: (1) how often an interaction occurred in models that improved the base model (frequency), and (2) the information content of the best model with this interaction (score), using the Akaike information criterion to penalize for additional interactions/parameters (Turkheimer et al., 2003). To further distill and condense the information from our millions of simulations into one metric and ranking the different metabolite-enzyme interactions, we used rank product analysis as a non-parametric statistical method (Messiha et al., 2014). This method ranks every interaction based on the geometric mean of the individual rank achieved in frequency and score (Transparent Methods), revealing G6P and 6PG dehydrogenase in the oxidative PP pathway as the main targets of regulation with glycolytic phosphofructokinase following in second place, the color of the heatmap showing the rank, and therefore importance, of each interaction (Figure 5A). Our results clearly demonstrate that, in every tested experiment, with the exception of the high stress level for *S. cerevisiae*, the interaction that was consistently the best was the NADPH inhibition of G6P dehydrogenase (Figures 5A and 5B). We therefore validated the previously suggested NADPH inhibition for *E. coli* (Christodoulou et al., 2018) and human dermal fibroblast G6P dehydrogenase (Kuehne et al., 2015) and demonstrated physiological relevance for the reported *in vitro* inhibition of the *S. cerevisiae* enzyme (Llobell et al., 1988) (Figure S18).

The only case in which NADPH inhibition of G6P dehydrogenase does not appear to play an important role *in vivo* was the high oxidative stress challenge in *S. cerevisiae*. Although ROS blockage of the lower glycolytic enzyme GAP dehydrogenase (Ralser et al., 2009) was particularly important for this condition (Figure S16), the increase in metabolite S7P (Figure S17) cannot be captured by inhibition of GAP dehydrogenase alone (see Figures S16 and S17). The putative interaction that explains these results (which could not be validated *in vitro*) was relief of G6P dehydrogenase by pyruvate inhibition, which at the functional level could achieve a similarly rapid increase in oxidative PP pathway flux as NADPH inhibition.

## Discussion

By combining metabolomics with multivariate analysis, timing analysis, and computational modeling, we revealed a striking conservation of the metabolic response to oxidative stress and the underlying metabolite-protein interactions in the widely different species *E. coli, S. cerevisiae*, and human dermal fibroblasts. This is surprising because previously different mechanisms were suggested to mediate rapid responses that mitigate the stress implications and stabilize the cellular redox potential in different species. The main regulatory interaction that achieves the rapid flux rerouting into the oxidative PP pathway for NADPH regeneration is the relief of G6P dehydrogenase from NADPH inhibition. Although it was known that this allosteric interaction occurs in most kingdoms of life (Reznik et al., 2017), we demonstrate here that it is the main mechanistic basis for a widely conserved metabolic response. The sole exception was the high oxidative stress in yeast, where the rapid increase in oxidative PP pathway flux was achieved by relief from pyruvate inhibition. Mechanistically, the relief of G6P dehydrogenase from NADPH inhibition may be achieved by competition for the active side or allosteric interaction, or a combination of both.

Alleviation of G6P dehydrogenase from inhibition implies that all three cell types do not use the full flux capacity of the oxidative PP pathway enzymes during normal growth. Consistently, maximum *in vitro* enzyme activities of G6P dehydrogenase (Sauer et al., 2004, Fuhrer et al., 2005, Ralser et al., 2007) are about 2-fold higher in *E. coli* and *S. cerevisiae* than the *in vivo* determined fluxes through the oxidative PP pathway (Fuhrer et al., 2005, Park et al., 2016). In mammalian cells the *in vitro* activity is even 40 times higher (Table S3). This investment into a reserve flux capacity enables an immediate metabolic response and thereby contributes to an intrinsic tolerance against oxidative stress, as was demonstrated for *E. coli* (Christodoulou et al., 2018). Our results do not provide any evidence for a function of ROS inhibition of GAP dehydrogenase. It can cause specific dynamics of glycolytic metabolites such as PEP and DHAP but does not appear to have a major functional role in increasing the PP pathway flux. Although our results strongly suggest that not only the response but also the molecular implementation is conserved across kingdoms, we cannot exclude that additional metabolite-enzyme interactions, beyond those evaluated here, may be important, both in the cell lines tested and other organisms. Indeed, Ralser et al. (2007) observed that changes in enzymatic activity of *S. cerevisiae* triose-phosphate isomerase and pyruvate kinase increased concentrations of PP pathway intermediates, suggesting a possible interplay between lower glycolysis and the PP pathway.

### Limitations of the Study

We see three main limitations of our study that point to potential caveats. The first limitation regards the decrease of succinate levels across all species. The inconsistencies observed in few cases, where succinate is not consistently decreasing in all cases, could be explained by a potential direct conversion of aKG to succinate to neutralize ROS (Liu et al., 2018). The second limitation is the lack of instantaneous measurements of glucose uptake rate immediately after the oxidative stress treatment, which is exceptionally challenging to quantify at a second scale. Hence, we cannot exclude changes in glucose uptake, but such putative changes would not affect our conclusion because our earlier ^13^C labeling experiments at a second resolution (Christodoulou et al., 2018, Kuehne et al., 2015) demonstrated a flux ratio shift toward the oxidative PP pathway upon oxidative stress. Thus, even if the glucose uptake would change, there would still be a relatively higher flux through the PP pathway. Furthermore, we tested *in silico*, with our ensemble modeling framework, models where regulation would directly affect (increase or decrease) the glucose uptake rate. These models always scored extremely low, suggesting that such instantaneous changes in the glucose uptake rate are not likely to be the cause of the system's dynamic behavior. The third limitation concerns the precise mechanism of G6PDH inhibition by NADPH that may be competitive, allosteric, or a combination of both. We do have evidence that NADPH inhibits G6PD by competing for the active site, at least in *E. coli* (Christodoulou et al., 2018); however, we cannot exclude the possibility of allosteric regulation as well.

## Methods

All methods can be found in the accompanying Transparent Methods supplemental file.

## Supplemental Information

Document S1. Transparent Methods, Figures S1–S18, and Tables S2 and S3

Table S1. Overview of Normalized Non-targeted Metabolomics Data, Related to Figures 2, 3, and 4

Table S4. Results of the Model-Based Identification of Mechanisms that Regulate the Metabolic Response to Oxidative Stress in *E. coli* Treated with 0.5 mM H_2_O_2_, Related to Figure 5

Table S5. Results of the Model-Based Identification of Mechanisms that Regulate the Metabolic Response to Oxidative Stress in *E. coli* Treated with 20 mM H_2_O_2_, Related to Figure 5

Table S6. Results of the Model-Based Identification of Mechanisms that Regulate the Metabolic Response to Oxidative Stress in *S. cerevisiae* Treated with 0.5 mM H_2_O_2_, Related to Figure 5

Table S7. Results of the Model-Based Identification of Mechanisms that Regulate the Metabolic Response to Oxidative Stress in *S. cerevisiae* Treated with 20 mM H_2_O_2_, Related to Figure 5

Table S8. Results of the Model-Based Identification of Mechanisms that Regulate the Metabolic Response to Oxidative Stress in Human Dermal Fibroblasts Treated with 0.5 mM H_2_O_2_, Related to Figure 5

Table S9. Results of the Model-Based Identification of Mechanisms that Regulate the Metabolic Response to Oxidative Stress in Human Dermal Fibroblasts Treated with 20 mM H_2_O_2_, Related to Figure 5

Table S10. Overview of Aggregated Targeted Metabolomics Data Used for the Model-based Identification of Mechanisms that Regulate the Metabolic Response to Oxidative Stress, Related to Figure 5
