# Supplementary material for: Reserve Flux Capacity in the Pentose Phosphate Pathway by NADPH Binding Is Conserved across Kingdoms
Source: iScience. 2019 Aug 29;19:1133–44. doi: 10.1016/j.isci.2019.08.047 (PMC6831883; doi:10.1016/j.isci.2019.08.047)
Supplement: Document S1. Transparent Methods, Figures S1–S18, and Tables S2 and S3 [file mmc1.pdf]

ISCI, Volume *19*

Supplemental Information

Reserve Flux Capacity in the Pentose Phosphate Pathway by NADPH Binding Is Conserved across Kingdoms

Dimitris Christodoulou, Andreas Kuehne, Alexandra Estermann, Tobias Fuhrer, Paul Lang, and Uwe Sauer

Transparent Methods

Kinetic Model of Glycolysis/Gluconeogenesis and the Pentose Phosphate Pathway

The irreversible reactions and transport of glucose are described by Michaelis-Menten kinetics:

$$v = v_{max} \frac{c_{Substrate}}{c_{Substrate} + K_M}$$

Similar to previous studies(Link, Kochanowski and Sauer, 2013) we assume that reversible reactions are near equilibrium and the law of mass action describes the kinetics for the forward (+) and backward (-) direction in these cases.

$$v^+ = k^+ c_{Substrate}$$

$$v^- = k^- c_{Product}$$

The detailed equations are given below:

Kinetic rate equations – irreversible reactions:
Reaction 1

glucose specific phosphotransferase system

$$v_{PTS} = v_{max,PTS} \frac{c_{Glucose}}{c_{Glucose} + K_{PTS,Glucose}}$$

Reaction 2

phosphofructokinase (PFK)

$$v_{PFK} = v_{max,PFK} \frac{c_{F6P}}{c_{F6P} + K_{PFK,F6P}}$$

Reaction 3

fructose-1,6-bisphosphatase (FBPase)

$$v_{FBPase} = v_{max,FBPase} \frac{c_{FBP}}{c_{FBP} + K_{FBPase,FBP}}$$

Reaction 4

glucose-6-phosphatedehydrogenase (G6PDH)

$$v_{G6PDH} = v_{max,G6PDH} \frac{c_{G6P}}{c_{G6P} + K_{G6PDH,G6P}}$$

Reaction 5

6-phosphogluconate dehydrogenase (GND)

$$v_{GND} = v_{max,GND} \frac{c_{6PG}}{c_{6PG} + K_{GND,6PG}}$$

Reaction 6

pyruvate kinase (PYK)

$$v_{PYK} = v_{max,PYK} \frac{c_{PEP}}{c_{PEP} + K_{PYK,PEP}}$$

Reaction 7

phosphoenolpyruvate synthetase (PPS)

$$v_{PPS} = v_{max,PPS} \frac{c_{PYR}}{c_{PYR} + K_{PPS,PYR}}$$

Reaction 8

pyruvate dehydrogenase (PDH)

$$v_{PDH} = v_{max,PDH} \frac{c_{PYR}}{c_{PYR} + K_{PDH,PYR}}$$

Reaction 9

phosphoenolpyruvate carboxylase (PPC)

$$v_{PDH} = v_{max,PDH} \frac{c_{PEP}}{c_{PEP} + K_{PDH,PEP}}$$

Kinetic rate equations – reversible reactions:
Reaction 10/11

phosphoglucoseisomerase (PGI)

$$v_{PGI}{}^{+} = k_{PGI}^{+} c_{G6P}$$

$$v_{PGI}{}^{-} = k_{PGI}^{-} c_{F6P}$$

Reaction 12/13

fructose-1,6-bisphosphate aldolase (ALD).

Instead of GAP and DHAP this reaction produces 2 molecules DHAP, since we assume that GAP and DHAP are in equilibrium by triose phosphate isomerase.

$$v_{ALD}{}^{+} = k_{ALD}^{+} c_{FBP}$$

$$v_{ALD}{}^{-} = k_{ALD}^{-} c_{DHAP} c_{DHAP}$$

Reaction 14/15

glyceraldehyde-3-phosphate dehydrogenase (GAPDH), phosphoglucokinase (PGK), phosphoglucomutase (PGM) and enolase (ENO) are in equilibrium(Link, Kochanowski and Sauer, 2013) and lumped into one reaction.

$$v_{GAPDH}{}^{+} = k_{GAPDH}^{+} c_{DHAP}$$

$$v_{GAPDH}{}^{-} = k_{GAPDH}^{-} c_{PEP}$$

Reaction 16/17

transketolase A (TKTA)

$$v_{TKTA}{}^{+} = k_{TKTA}^{+} c_{P5P}$$

$$v_{TKTA}{}^{-} = k_{TKTA}^{-} c_{DHAP} c_{S7P}$$

Reaction 18/19

transketolase B (TKTB)

$$v_{TKTB}{}^{+} = k_{TKTB}^{+} c_{P5P} c_{E4P}$$

$$v_{TKTB}{}^{-} = k_{TKTB}^{-} c_{DHAP} c_{F6P}$$

Reaction 20/21

transaldolase (TALA)

$$v_{TALA}{}^{+} = k_{TALA}^{+} c_{DHAP} c_{S7P}$$

$$v_{TALA}{}^{-} = k_{TALA}^{-} c_{E4P} c_{F6P}$$

Reaction 22

biosynthetic E4P drain (E4PD)

$$v_{E4PD}{}^{+} = k_{E4PD}^{+} c_{E4P}$$

Reaction 23

biosynthetic P5P drain (P5PD)

$$v_{P5PD}{}^{+} = k_{P5PD}^{+} c_{P5P}$$

Reaction 24

anabolic proxy of NADPH drain (NADPHD)

$$v_{NADPHD}{}^{+} = k_{NADPHD}^{+} c_{NADPH}$$

Reaction 25

generation of ROS from external source (ROSG)

this reaction is implemented as a constant input (that we vary in the different simulations)

$$v_{ROSG}{}^{+} = k^{+}_{ROSG}$$

Reaction 26

scavenging of ROS with NADPH (ROSS)

$$v_{ROSS}{}^{+} = k^{+}_{ROSS} c_{ROS}$$

Kinetic rate equations: Small molecule – enzyme interactions

An interaction between an enzyme catalyzing reaction *i* and a small molecule *j* is included as a power law term affecting the reaction rate.

$$v_i{}^{*} = v_{max,i} \prod_j \left(\frac{c_j}{c_{j,0}}\right)^{a_{i,j}}$$

In the base model without interactions (except the one from ROS on GAPDH), all exponents $a_{i,j}$ are zero and therefore the power law terms equal to 1. With this model we managed to easily search the topological space by testing ensembles of structurally different models by setting the according exponent to real-valued numbers. With this approach we also managed to create a parallel algorithm that does so efficiently.

Ordinary Differential Equations (ODEs)

$$\frac{dG6P}{dt} = Reaction1 - Reaction4 - Reaction10 + Reaction11 =$$
$$= v_{max,PTS} \frac{c_{Glucose}}{c_{Glucose} + K_{PTS,Glucose}} - v_{max,G6PDH} \frac{c_{G6P}}{c_{G6P} + K_{G6PDH,G6P}} - k^{+}_{PGI} c_{G6P} + k^{-}_{PGI} c_{F6P}$$

$$\frac{dF6P}{dt} = -Reaction2 + Reaction3 + Reaction10 - Reaction11 + Reaction18 - Reaction19 + Reaction20 - Reaction21$$

$$\frac{dFBP}{dt} = Reaction2 - Reaction3 - Reaction12 + Reaction13$$

$$\frac{dDHAP}{dt} = 2 \cdot Reaction12 - 2 \cdot Reaction13 - Reaction14 + Reaction15 + Reaction16 - Reaction17 + Reaction18 - Reaction19 - Reaction20 + Reaction21$$

$$\frac{d6PG}{dt} = Reaction4 - Reaction5$$

$$\frac{dPEP}{dt} = -Reaction1 - Reaction6 + Reaction7 - Reaction9 + Reaction14 - Reaction15$$

$$\frac{dPYR}{dt} = Reaction1 + Reaction6 - Reaction7 - Reaction8$$

$$\frac{dP5P}{dt} = Reaction5 - 2 \cdot Reaction16 + 2 \cdot Reaction17 - Reaction18 + Reaction19 - Reaction23$$

$$\frac{dE4P}{dt} = -Reaction18 + Reaction19 + Reaction20 - Reaction21 - Reaction22$$

$$\frac{dS7P}{dt} = Reaction16 - Reaction17 - Reaction20 + Reaction21$$

$$\frac{dNADPH}{dt} = Reaction4 + Reaction5 - Reaction24 - Reaction26$$

$$\frac{dROS}{dt} = Reaction25 - Reaction26$$

Quantification And Statistical Analysis

Parameterization of the kinetic model of glycolysis/gluconeogenesis and the pentose phosphate pathway

Kinetic parameters followed from statistical sampling of unknown parameters and a steady analysis as described below.

K_M values

The K_M values were randomly sampled from an interval of 0.1-10 times the *in vitro* determined literature value.

Steady state analysis and statistical sampling

In order to determine V_{max} values we performed a steady state analysis using measured glucose uptake rate (1.17 mM/sec). Metabolic fluxes were estimated by flux balance analysis during growth on glucose (Fong, Marciniak and Palsson, 2003) and we considered uncertainties about the fluxes by taking into account measured flux distributions (Gerosa *et al.*, 2015) and by statistical sampling of 5 parameters:

1. **Futile cycling between PFK and FBPase**: $\frac{v_{FBPase,0} - v_{PFK,0}}{v_{FBPase,0}} = 0 - 1$
2. **Futile cycling between PYK and PPS**: $\frac{v_{PPS,0} - v_{PYK,0}}{v_{PPS,0}} = 0 - 1$
3. **PP pathway flux**: 15-40% of the glucose uptake
4. **Biosynthetic drain of pentoses (P5P) and E4P**: 50-70% of the PP pathway flux
5. **Immediate increase in ROS flux**: 0.5 – 1

The steady state reaction rates (v^0) of all reactions follow from these unknown flux ratios and the measured glucose rate. Subsequently, the V_{max} of reaction *i* follows from v_i^0, the sampled $K_{i,M}$ and the measured steady state concentrations c_j^0 of the particular small molecule:

$$v_{i,max} = v_i^0(1 + \frac{K_{i,M}}{c_j^0})$$

Rate constants of reversible reactions

In the case of reversible reactions, we statistically sample for every pair (e.g. reactions 10/11) the efficiency of this reaction: if we know that a glycolytic flux of 1 goes through this reaction pair in the glycolytic direction and the efficiency is 0.5, this means that reaction 10 will have a flux of 2 and reaction 11 a flux of 1. The rate constants k^+ and k^- are calculated following this approach.

Selection of the best parameter set for each model topology

As described in the main text, we randomly sampled P = 20000 for each of the models with single small-molecule enzyme interactions and P = 2000 for each of each model with pairs of small-molecule enzyme interactions. For each parameter set, the simulation was performed with MATLAB. The residuals between the simulated species (indicated by ') and the measured species are calculated at t=5 time points for s = 8 species where we have absolute concentrations for. Due to differences in the absolute metabolite concentrations we estimated the sum of squared errors for s=8 relative metabolite concentrations ($\tilde{c}$) that are normalized to the glucose steady state concentrations:

$$SSR_c = \sum_{s=1}^{8} \sum_{t=1}^{5} \left(\widetilde{c'_{s,t}} - \widetilde{c_{s,t}}\right)^2$$

We used this objective to select the best parameter set for each model topology.

Akaike Information Criterion (AIC)

In order to compare in a systematic manner the simulation results of models with different topologies and different number of parameters *K* (due to different numbers of small-molecule – enzyme interactions), we utilized the Akaike Information Criterion (AIC) (Link, Kochanowski and Sauer, 2013)(Federico E. Turkheimer, Hinz and Cunningham, 2003).

$$AIC = Nlog\left(\frac{SSR}{N}\right) + 2K$$

where *N* I the total number of residuals. A particular model X with small molecule – enzyme interactions is ranked relative to the base model by the difference of AICs:

$$\Delta AIC_{ModelX} = AIC_{BaseModel} - AIC_{ModelX}$$

Product rank calculation

The ranks of the pairwise interactions based on how often the interaction appears in models with ΔAIC>0 (frequency) and the best ΔAIC that was achieved with a model including this interaction, were taken into account in the calculation of the product rank of the interactions.

The product rank of an interaction *i* is calculated as the geometric mean of the two individual ranks that a certain interaction has achieved.

Analysis of data

The analysis of the (experimental and simulated) data was performed using custom MATLAB (MathWorks) software. MATLAB was used for all simulations and the kinetic model was partly implemented using the SimBiology toolbox.

Parallel ensemble modelling framework

All the different model topologies are populated as different model objects in one master server and then are consequently sent to different CPU cores - workers for simulation, using the High Performance Computing service of ETH, containing over 29000 processor cores with a theoretical performance reaching over 1000 teraflops. Depending on how many CPU cores are available, the time of computation for all the simulations (which depend on the number of model topologies and the number of different parameter sets we test for every model, in our case 12000 topologies for each organism and 2000 parameter sets yield ~ 120 million simulations) scales accordingly. Once the simulations from the different CPU cores – workers are finished, the saved simulated results return to the master, where they are processed and analyzed thus yielding the rank of every interaction, based on the criteria we have set (frequency and score). All code was written in MATLAB and various functions from the Parallel Computing toolbox and the SimBiology toolbox were used.

Table S1: Overview of normalized non-targeted metabolomics data. (Additional File)

Table S2: Kinetic parameters of reactions in the glycolysis – PP pathway models of the three different organisms. Vmax of irreversible reactions are estimable parameters and no value is given. Non-applicable values indicate that this particular enzyme is non-existent or does not carry flux in this particular organism. Related to Figure 5

Reaction	Parameter	Value Range *E. coli*	Value Range *S. cerevisiae*	Value Range *H. sapiens*
**Irreversible Reactions**				
PFK	$V_{max,PFK}$	-	-	-
	$K_{PFK,F6P}$	(0.1-10) ▪ 0.16mM	(0.1-10) ▪ 0.058mM	(0.1-10) ▪ 0.0425mM
FBPase	$V_{max,FBPase}$	-	-	-
	$K_{FBPase,FBP}$	(0.1-10) ▪ 0.015mM	(0.1-10) ▪ 0.2mM	(0.1-10) ▪ 0.0022mM
G6PDH	$V_{max,G6PDH}$	-	-	-
	$K_{G6PDH,G6P}$	(0.1-10) ▪ 0.2mM	(0.1-10) ▪ 0.042mM	(0.1-10) ▪ 0.045mM
GND	$V_{max,GND}$	-	-	-
	$K_{GND,6PG}$	(0.1-10) ▪ 0.1mM	(0.1-10) ▪ 0.062mM	(0.1-10) ▪ 0.02mM
PYK	$V_{max,PYK}$	-	-	-
	$K_{PYK,PEP}$	(0.1-10) ▪ 0.31mM	(0.1-10) ▪ 0.281mM	(0.1-10) ▪ 0.2mM
PPS	$V_{max,PPS}$	-	-	-
	$K_{PPS,PYR}$	(0.1-10) ▪ 0.083mM	Non-applicable	Non-applicable
PDH	$V_{max,PDH}$	-	-	-
	$K_{PDH,PYR}$	(0.1-10) ▪ 0.515mM	(0.1-10) ▪ 0.65mM	(0.1-10) ▪ 0.01mM
PPC	$V_{max,PPC}$	-	-	-
	$K_{PPC,PEP}$	(0.1-10) ▪ 0.19mM	(0.1-10) ▪ 0.08mM	Non-applicable

Table S3: In vivo activity of the enzyme G6P dehydrogenase under growth on glucose for the three different organisms. In vitro enzymatic activity of the same enzyme in all three organisms.

	E.coli		**Yeast**		**Mammalian cells**	
	in vivo (mmol/g/h)	in vitro (mmol/g/h)	in vivo (mM/min)	in vitro (mM/min)	in vivo (mM/min)	in vitro (mM/min)
**G6PDH**	**2**	**3.2**	**2**	**4**	**0.05**	**2.316**

***E. coli* in vitro**: (Fuhrer and Sauer, 2009), ***E. coli* in vivo**: (Fuhrer, Fischer and Sauer, 2005; Park *et al.*, 2016), **Yeast in vitro**: (Ralser *et al.*, 2007), **Yeast in vivo**: (Park *et al.*, 2016), **Mammalian cells in vitro**: BioNumbers, Mammalian G6P dehydrogenase Kinetics, Privately collected by Professor Armindo Salvador, **Mammalian cells in vivo**: (Park *et al.*, 2016)

Table S4: Results of the model-based identification of mechanisms that regulate the metabolic response to oxidative stress in *E. coli* treated with 0.5 mM H_2O_2. Related to Figure 5 (Additional File)

Table S5: Results of the model-based identification of mechanisms that regulate the metabolic response to oxidative stress in *E. coli* treated with 20 mM H_2O_2. Related to Figure 5 (Additional File)

Table S6: Results of the model-based identification of mechanisms that regulate the metabolic response to oxidative stress in *S. cerevisiae* treated with 0.5 mM H_2O_2. Related to Figure 5 (Additional File)

Table S7: Results of the model-based identification of mechanisms that regulate the metabolic response to oxidative stress in *S. cerevisiae* treated with 20 mM H_2O_2. Related to Figure 5 (Additional File)

Table S8: Results of the model-based identification of mechanisms that regulate the metabolic response to oxidative stress in human dermal fibroblasts treated with 0.5 mM H_2O_2. Related to Figure 5 (Additional File)

Table S9: Results of the model-based identification of mechanisms that regulate the metabolic response to oxidative stress in human dermal fibroblasts treated with 20 mM H_2O_2. Related to Figure 5 (Additional File)

Table S10: Overview of aggregated targeted metabolomics data used for the model-based identification of mechanisms that regulate the metabolic response to oxidative stress. Related to Figure 5 (Additional File)

Supplementary Figures

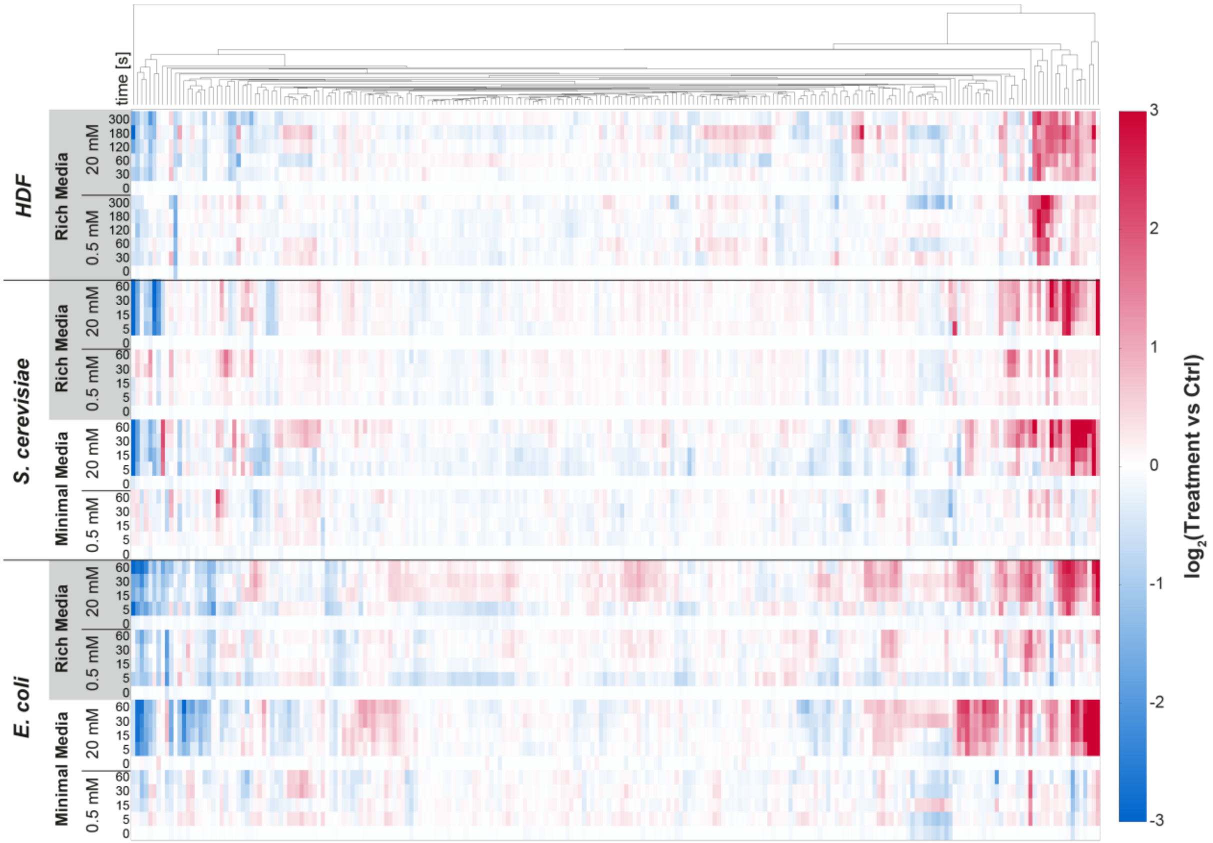

Figure S1: Overview of the normalized non-targeted metabolomics data. Heatmap shows mean values of metabolite data of treatments normalized to their respective controls. Related to Figure 2, 3, 4

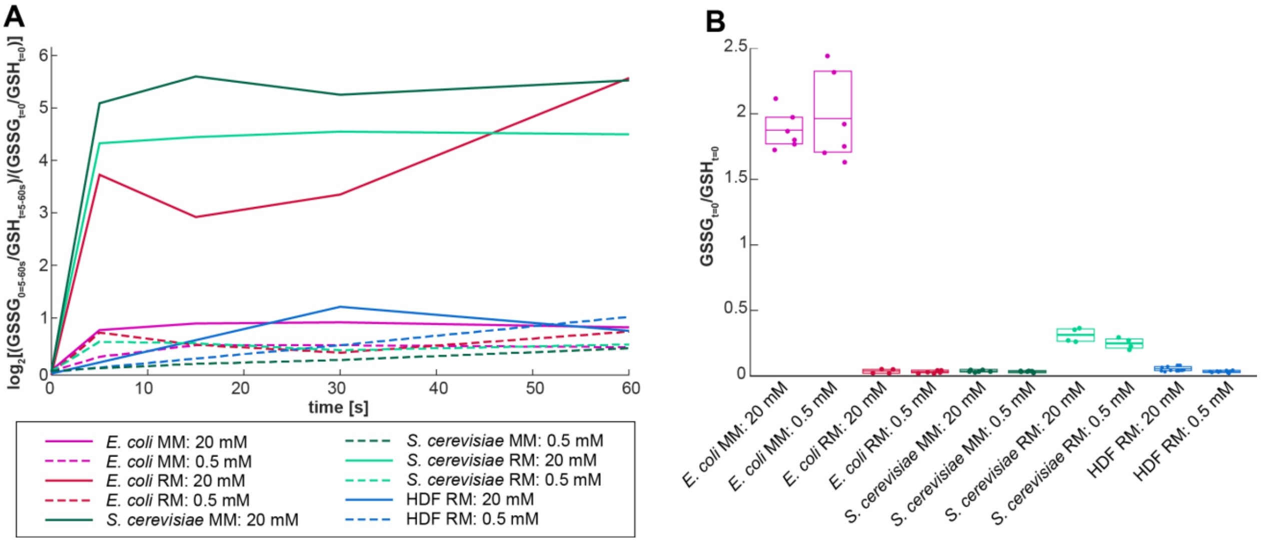

Figure S2: (A) Ratio of oxidized to reduced glutathione over time, after exposure to oxidative stress. (Note: The first measured treatment of H2O2 is after 30 seconds for every other organism after 5 seconds). **(B)** Ratio of oxidized to reduced glutathione in untreated samples. Related to section "The immediate metabolic response upon exposure to oxidative stress"

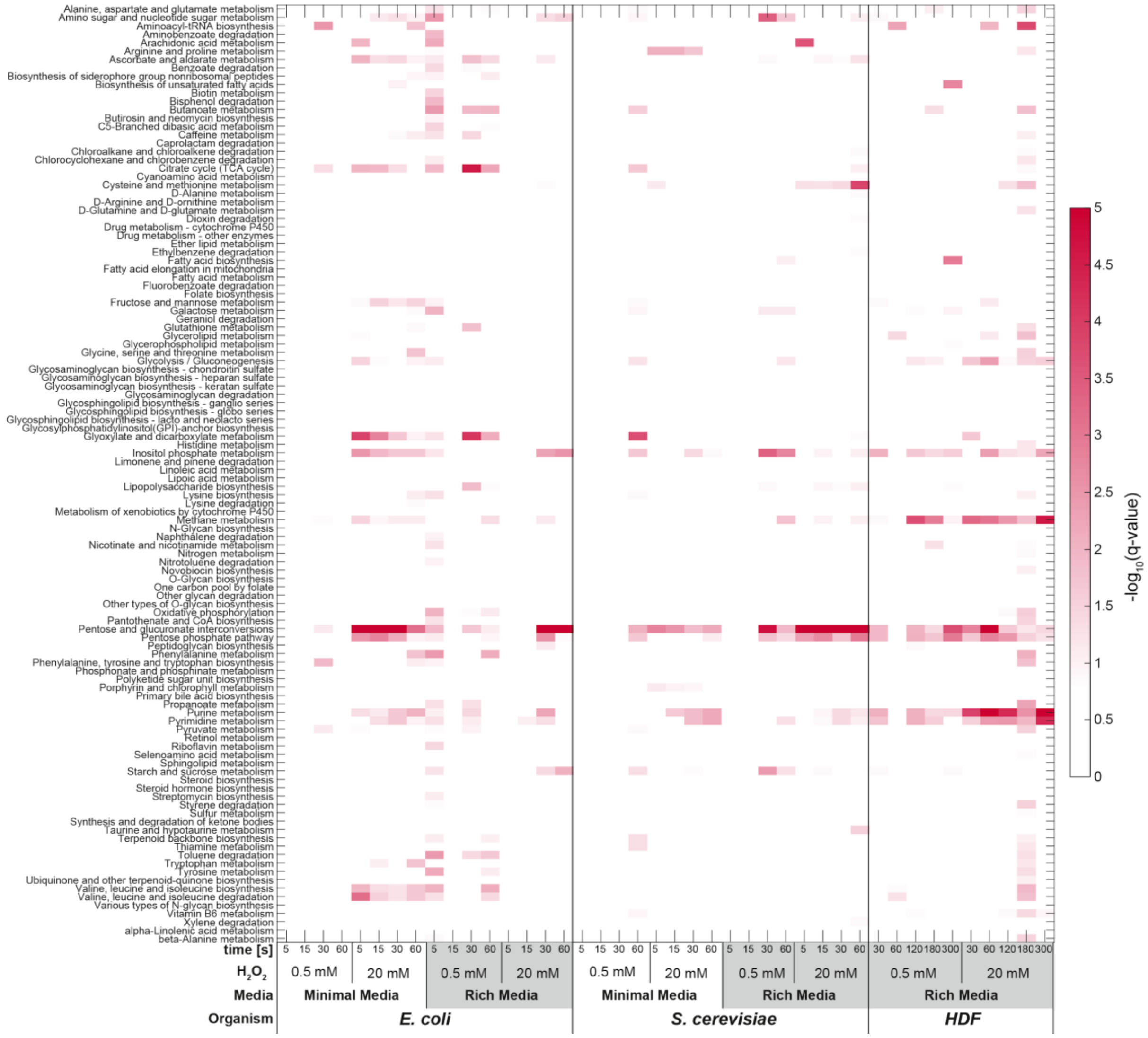

Figure S3: Pathway enrichment analysis on the measured metabolites changing at each time point, compared to untreated controls. Related to section "The immediate metabolic response upon exposure to oxidative stress"

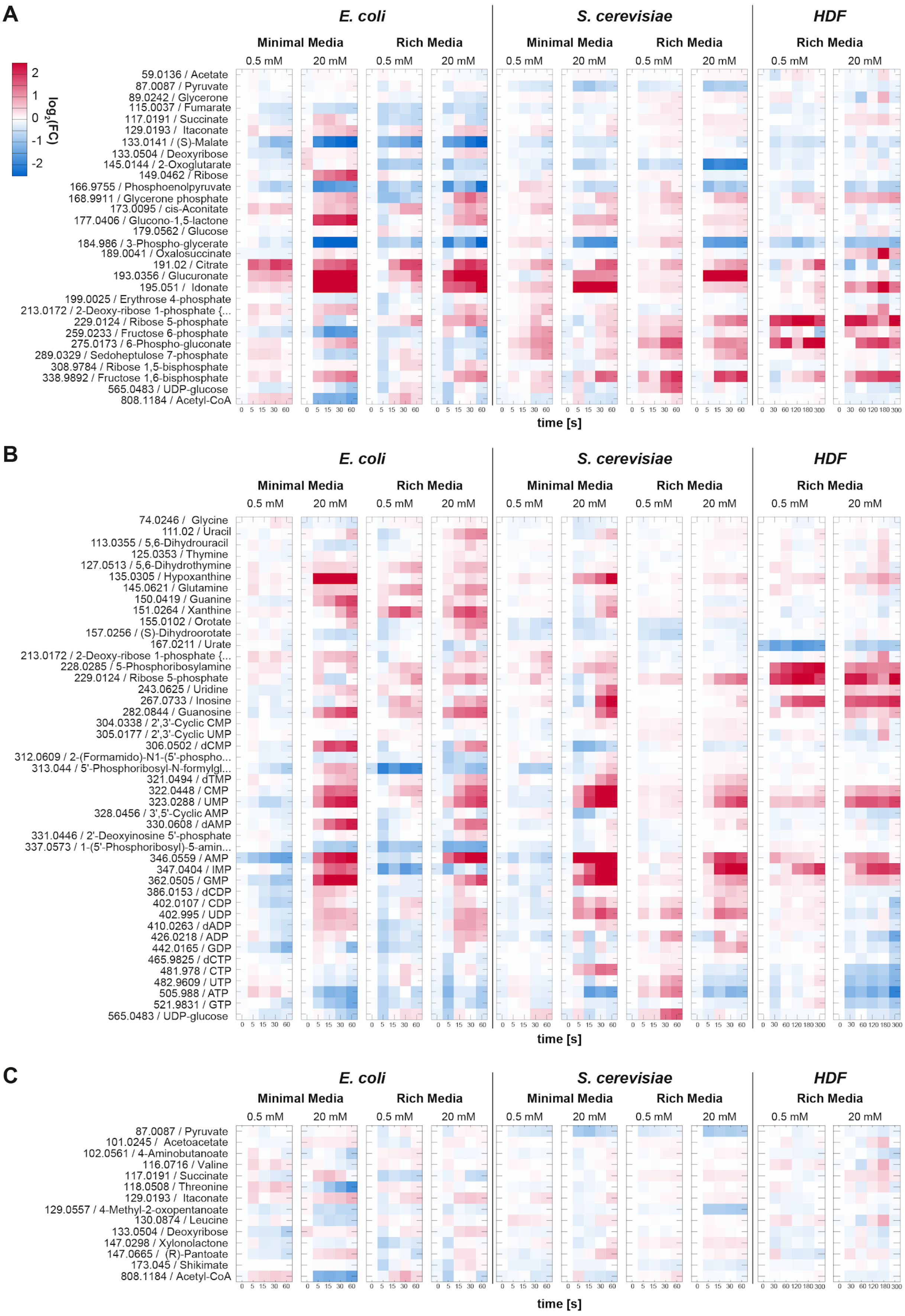

Figure S4: Relative metabolite changes in relevant pathways.: a) Glycolysis, PPP and TCA Cycle, b) purine and pyrimidine metabolism and c) Valine, Leucine, Isoleucine degradation and biosynthesis.

Heatmaps show mean values of relative metabolite changes log2(treatment vs control). Related to section "The immediate metabolic response upon exposure to oxidative stress"

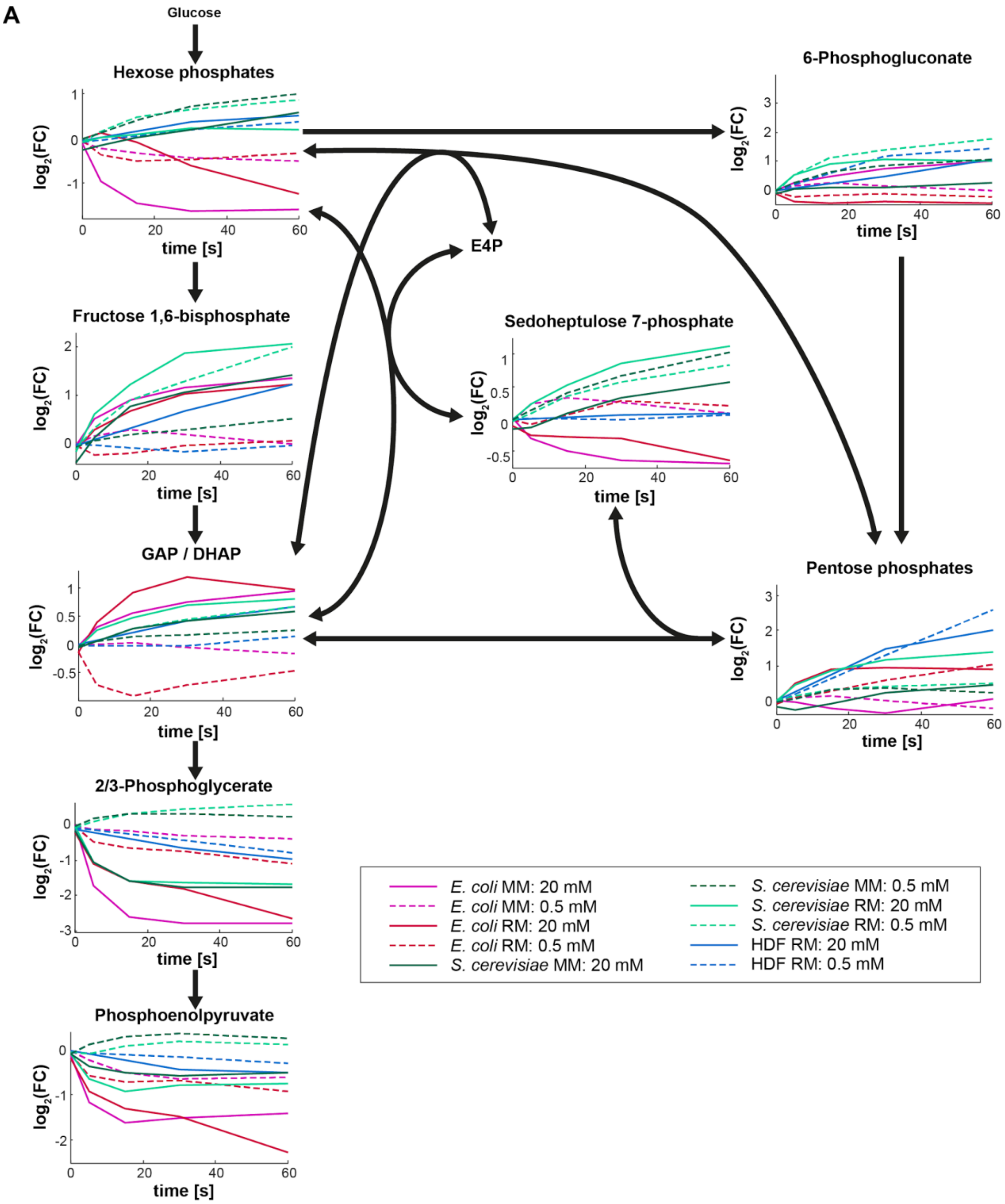

Figure S5: Metabolite Profiles of glycolytic and PP pathway intermediates, upon H_2O_2 treatment. The changes of each metabolite relative to the untreated condition (time point 0) are shown. Solid lines represent exposure to high stress (20 mM) while dashed lines represent exposure to low stress (0.5 mM)

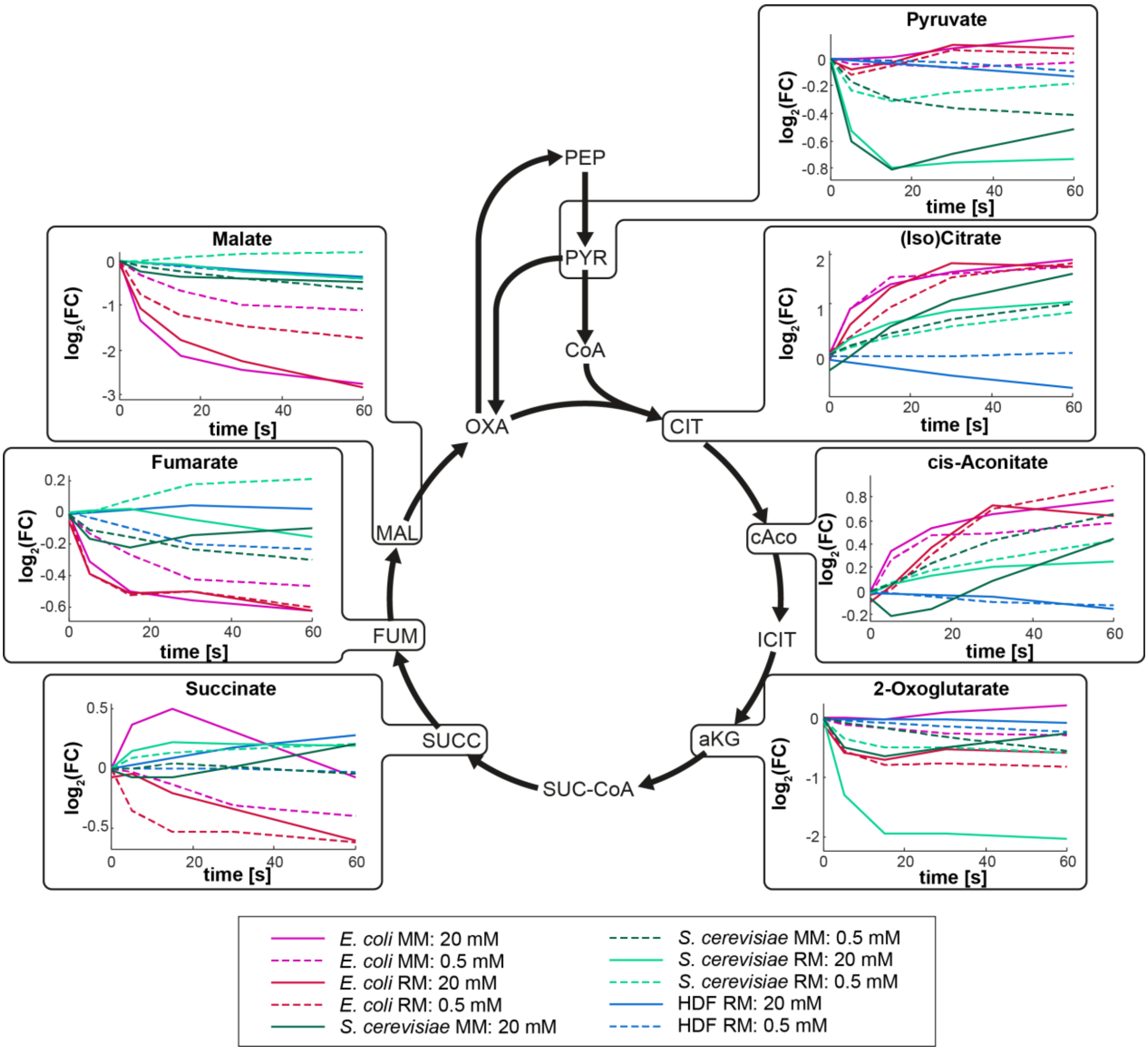

Figure S6: Metabolite profiles of citric acid cycle intermediates, upon H_2O_2 treatment. The changes of each metabolite relative to the untreated condition (time point 0) are shown. Solid lines represent exposure to high stress (20 mM) while dashed lines represent exposure to low stress (0.5 mM). Related to section "The immediate metabolic response upon exposure to oxidative stress"

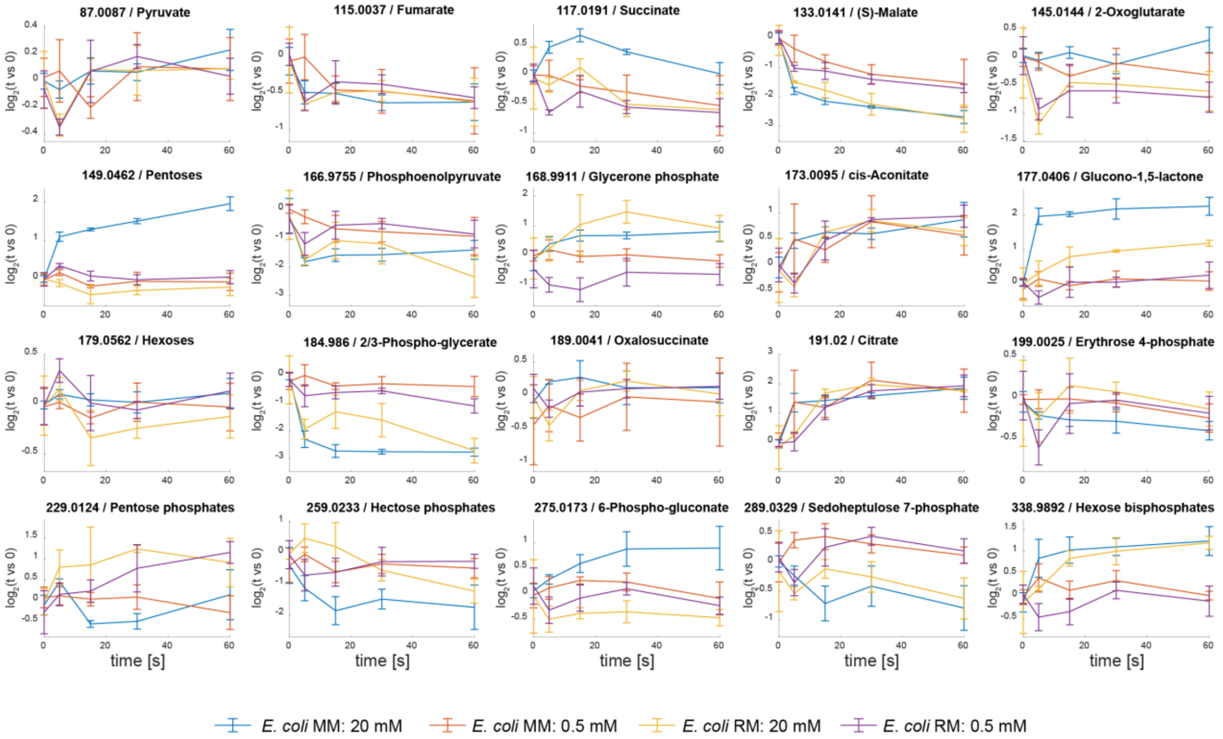

Figure S7: Metabolite Profiles of glycolytic and citric acid cycle intermediates of *E. coli*, upon H_2O_2 treatment. The changes of each metabolite relative to the untreated condition (time point 0) are shown. Plots show mean values +/- standard deviation of three biological replicates. Related to section "The immediate metabolic response upon exposure to oxidative stress"

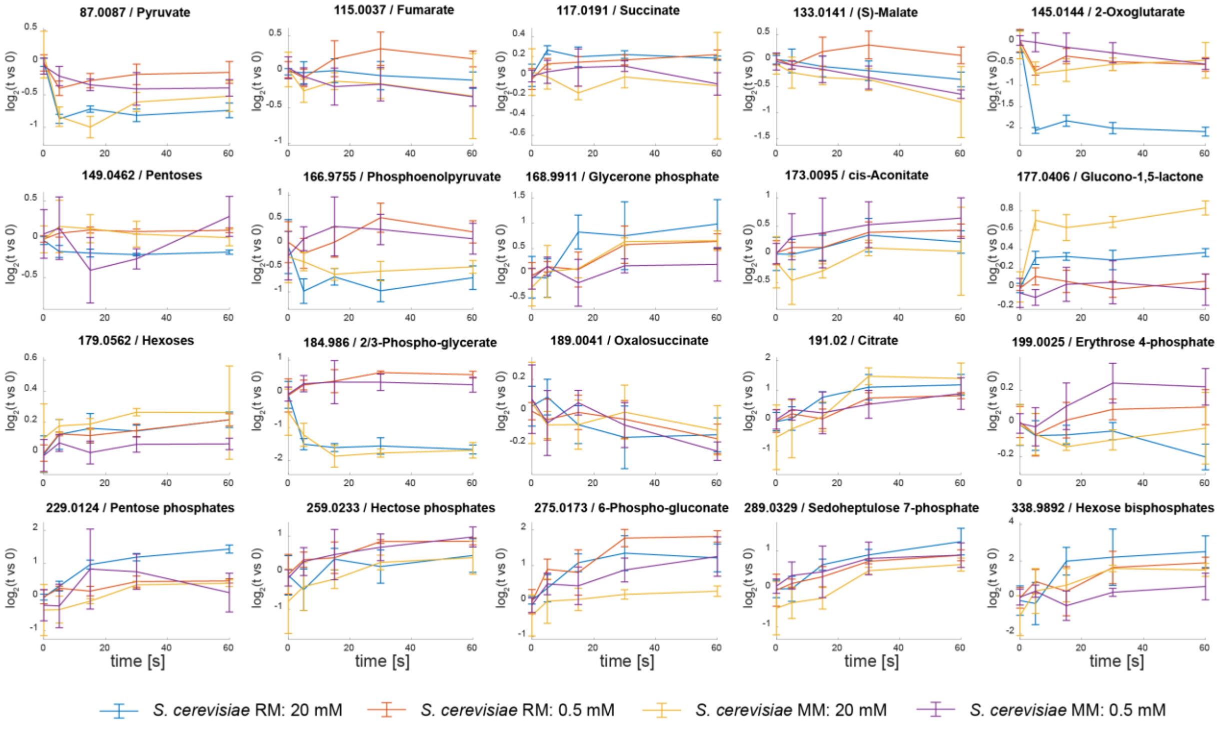

Figure S8: Metabolite Profiles of glycolytic and citric acid cycle intermediates of *S. cerevisiae*, upon H_2O_2 treatment. The changes of each metabolite relative to the untreated condition (time point 0) are

shown. Plots show mean values +/- standard deviation of three biological replicates. Related to section "The immediate metabolic response upon exposure to oxidative stress"

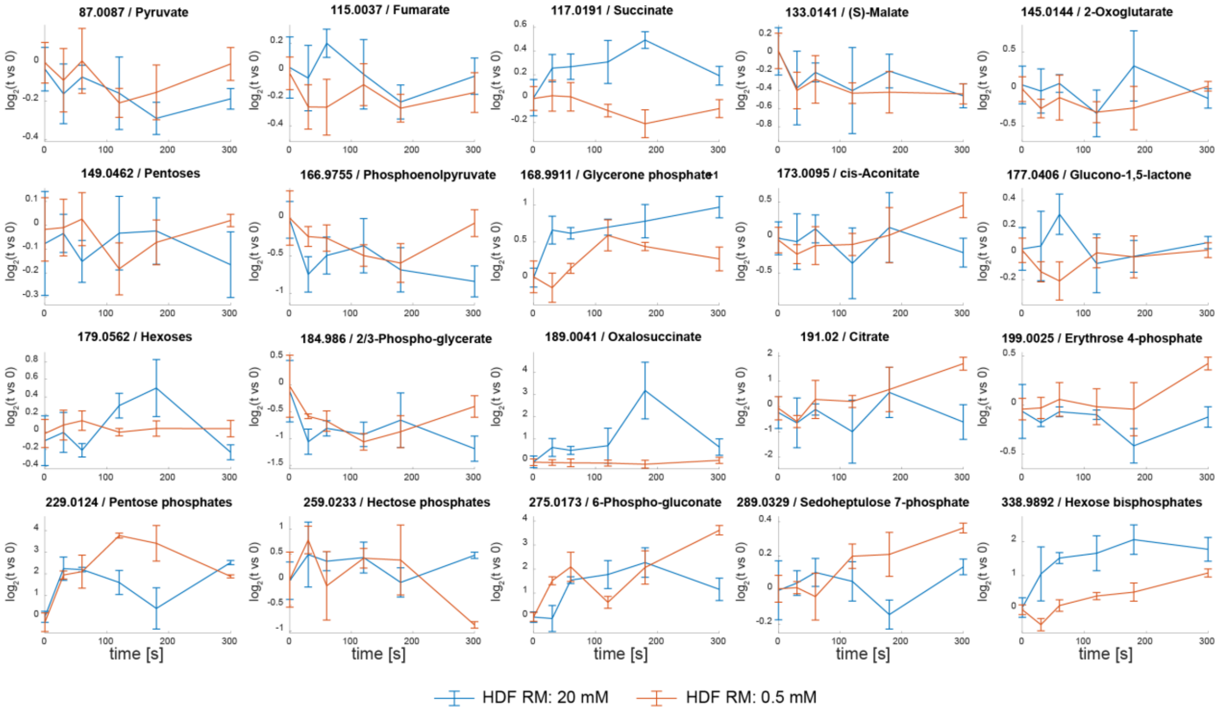

Figure S9: Metabolite Profiles of glycolytic and citric acid cycle intermediates of human dermal fibroblasts, upon H_2O_2 treatment. The changes of each metabolite relative to the untreated condition (time point 0) are shown. Plots show mean values +/- standard deviation of three biological replicates. Related to section "The immediate metabolic response upon exposure to oxidative stress"

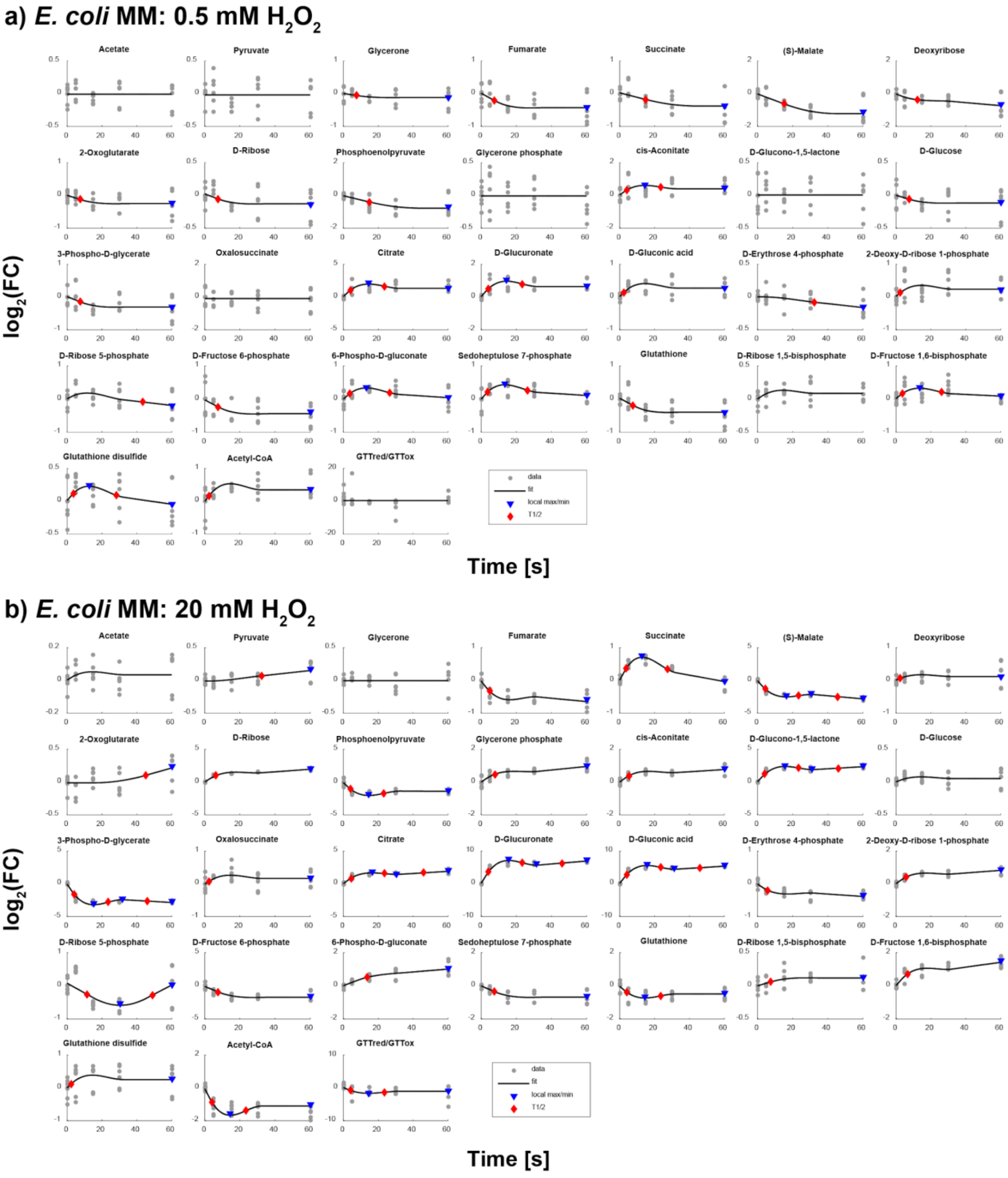

Figure S10 Fitting results of Multivariate Adaptive Regression Splines on metabolite traces of *E. coli* grown in minimal media and treated with a) 0.5 mM and b) 20 mM H_2O_2. Local maxima (including endpoints of treatment) were identified with a peak prominence of Δlog2(FC) > 0.2 were identified for fits with R2 < 0.2. Furthermore, following local maxima with less that 50% change of log2(FC) were removed. Related to Figure 3 and 4.

a) *E. coli* RM: 0.5 mM H_2O_2

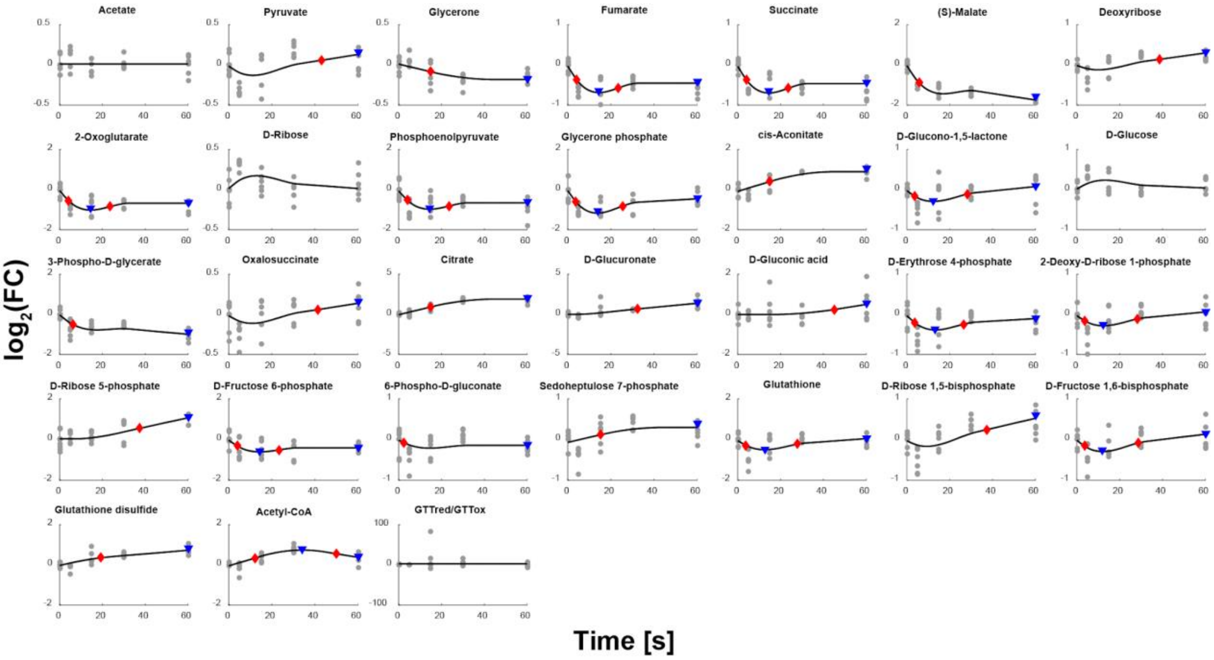

b) *E. coli* RM: 20 mM H_2O_2

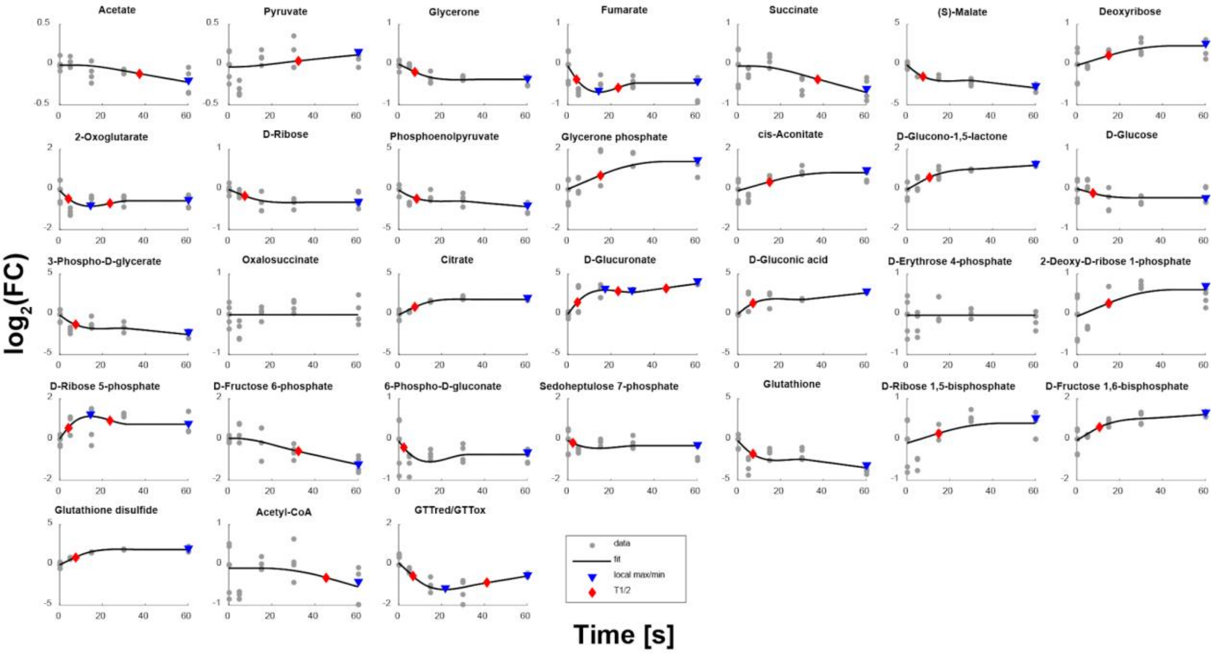

Figure S11 Fitting results of Multivariate Adaptive Regression Splines on metabolite traces of *E. coli* grown in rich media and treated with a) 0.5 mM and b) 20 mM H2O2. Local maxima (including endpoints of treatment) were identified with a peak prominence of Δlog2(FC) > 0.2 were identified for fits with R2 < 0.2. Furthermore, following local maxima with less that 50% change of log2(FC) were removed. Related to Figure 3 and 4.

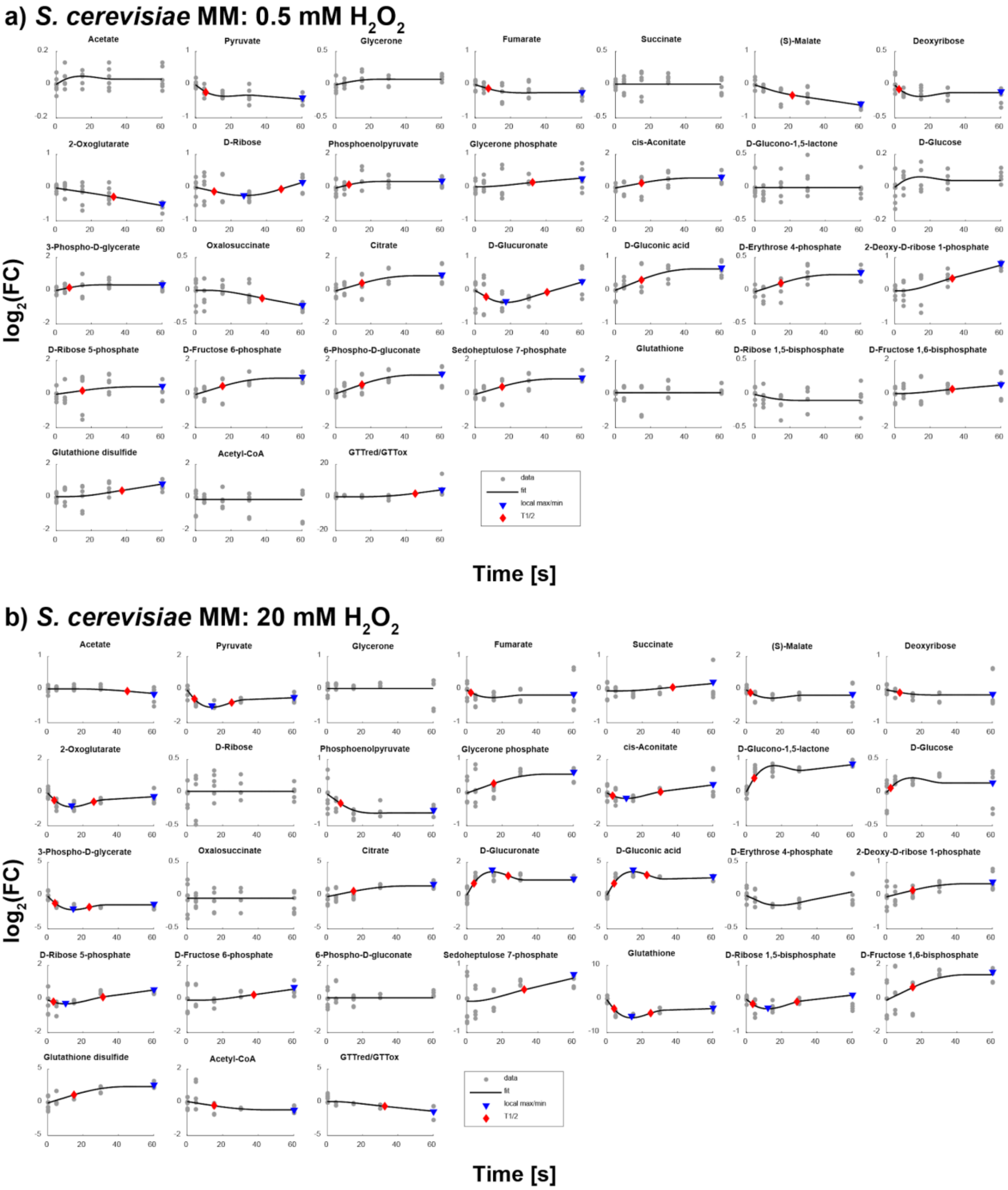

Figure S12 Fitting results of Multivariate Adaptive Regression Splines on metabolite traces of *S. cerevisiae* grown in minimal media and treated with a) 0.5 mM and b) 20 mM H2O2. Local maxima (including endpoints of treatment) were identified with a peak prominence of Δlog2(FC) > 0.2 were identified for fits with R2 < 0.2. Furthermore, following local maxima with less that 50% change of log2(FC) were removed. Related to Figure 3 and 4.

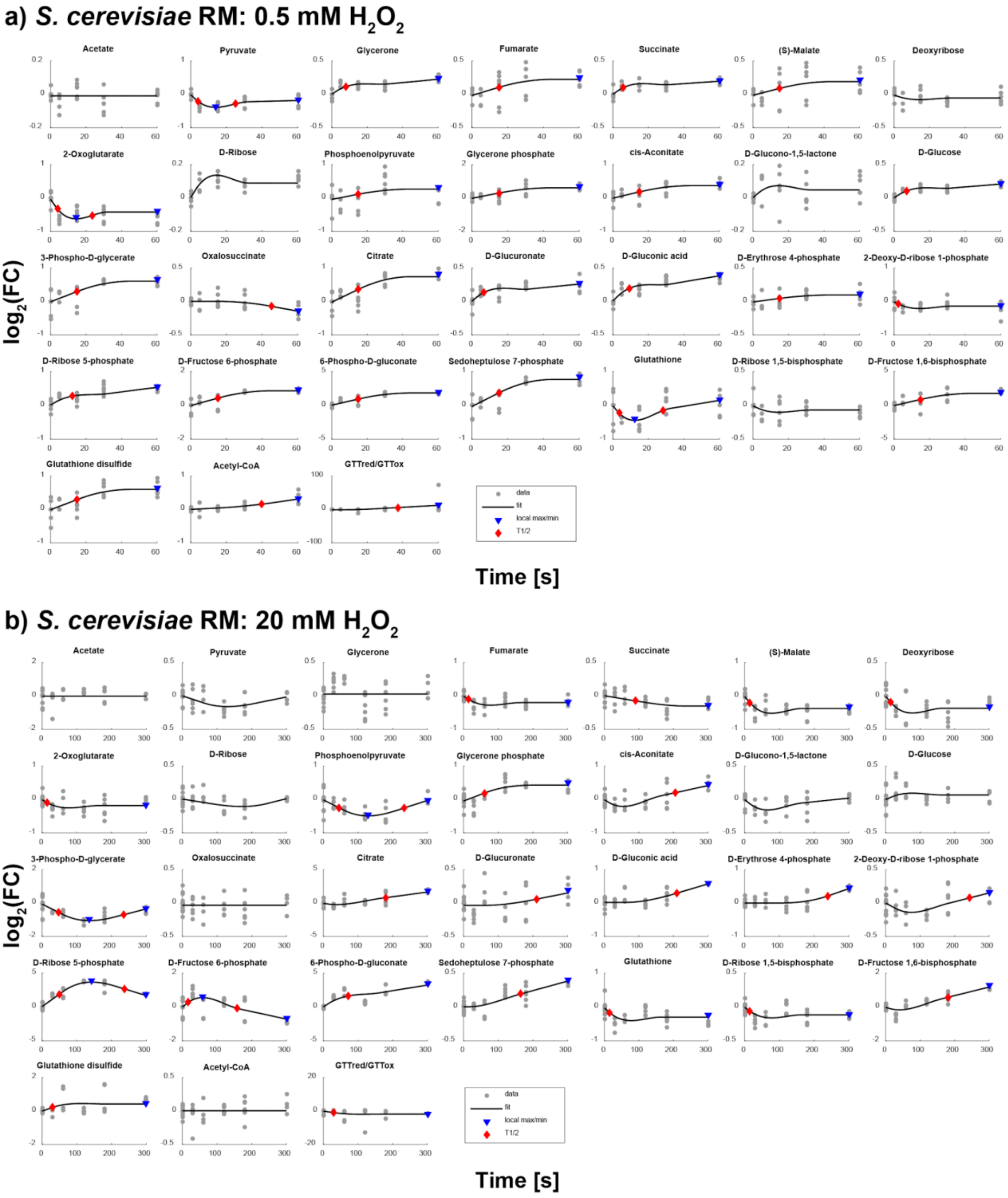

Figure S13 Fitting results of Multivariate Adaptive Regression Splines on metabolite traces of *S. cerevisiae* grown in rich media and treated with a) 0.5 mM and b) 20 mM H2O2. Local maxima (including endpoints of treatment) were identified with a peak prominence of Δlog2(FC) > 0.2 were identified for fits with R2 < 0.2. Furthermore, following local maxima with less that 50% change of log2(FC) were removed. Related to Figure 3 and 4.

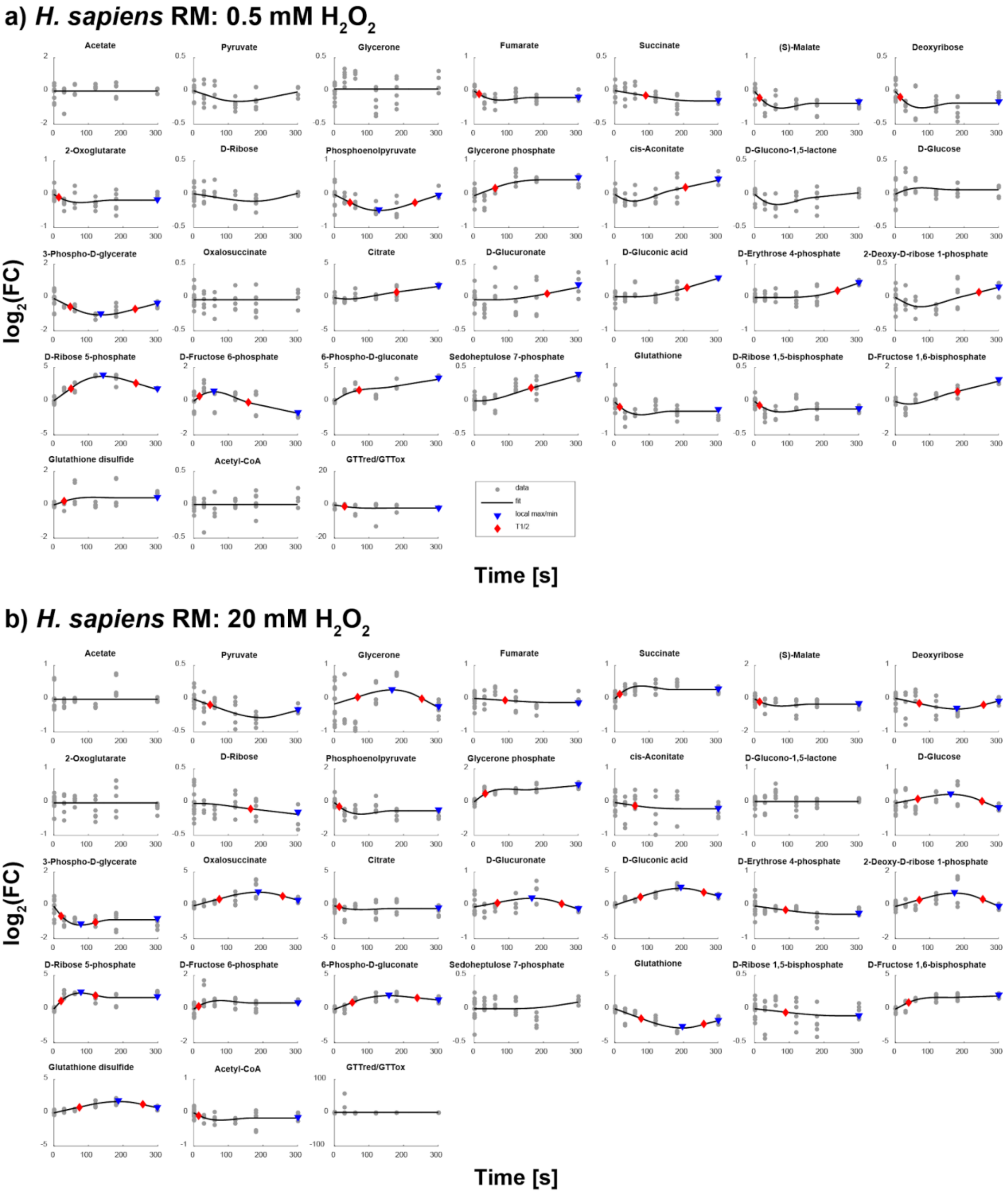

Figure S14 Fitting results of Multivariate Adaptive Regression Splines on metabolite traces of human dermal fibroblasts grown in rich media and treated with a) 0.5 mM and b) 20 mM H2O2. Local maxima (including endpoints of treatment) were identified with a peak prominence of Δlog2(FC) > 0.2 were identified for fits with R2 < 0.2. Furthermore, following local maxima with less that 50% change of log2(FC) were removed. Related to Figure 3 and 4.

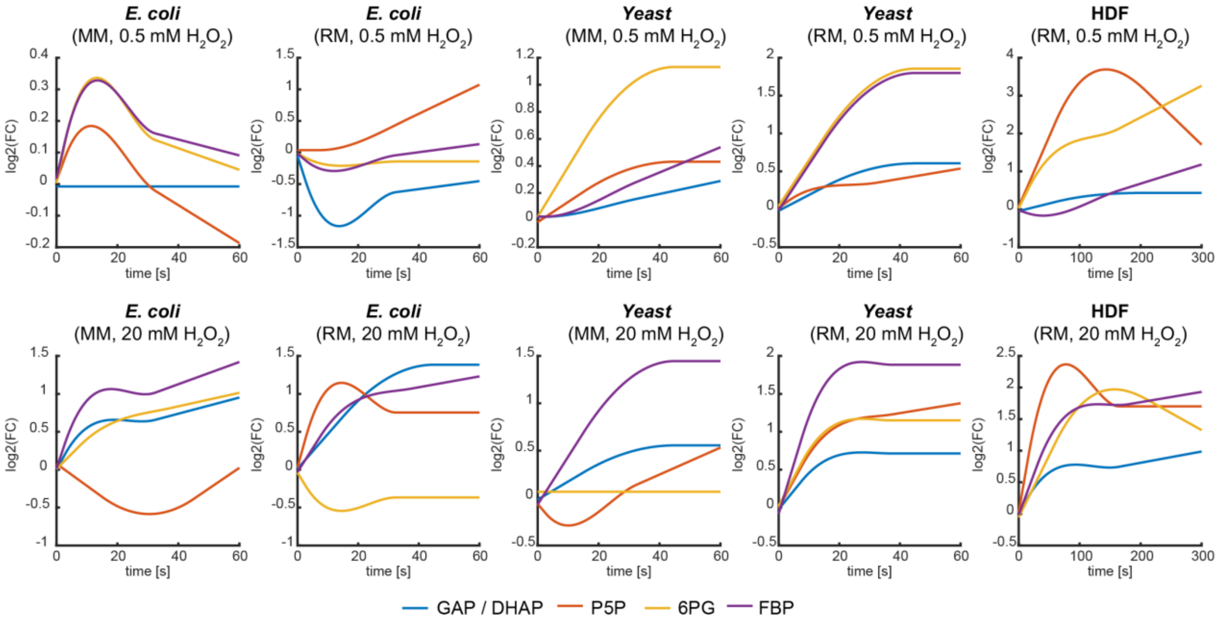

Figure S15 Comparison of the response of upper glycolytic and pentose phosphate pathway metabolites upon exposure to oxidative stress. Plots show the fitting results of Multivariate Adaptive Regression Splines. Related to Figure 3 and 4.

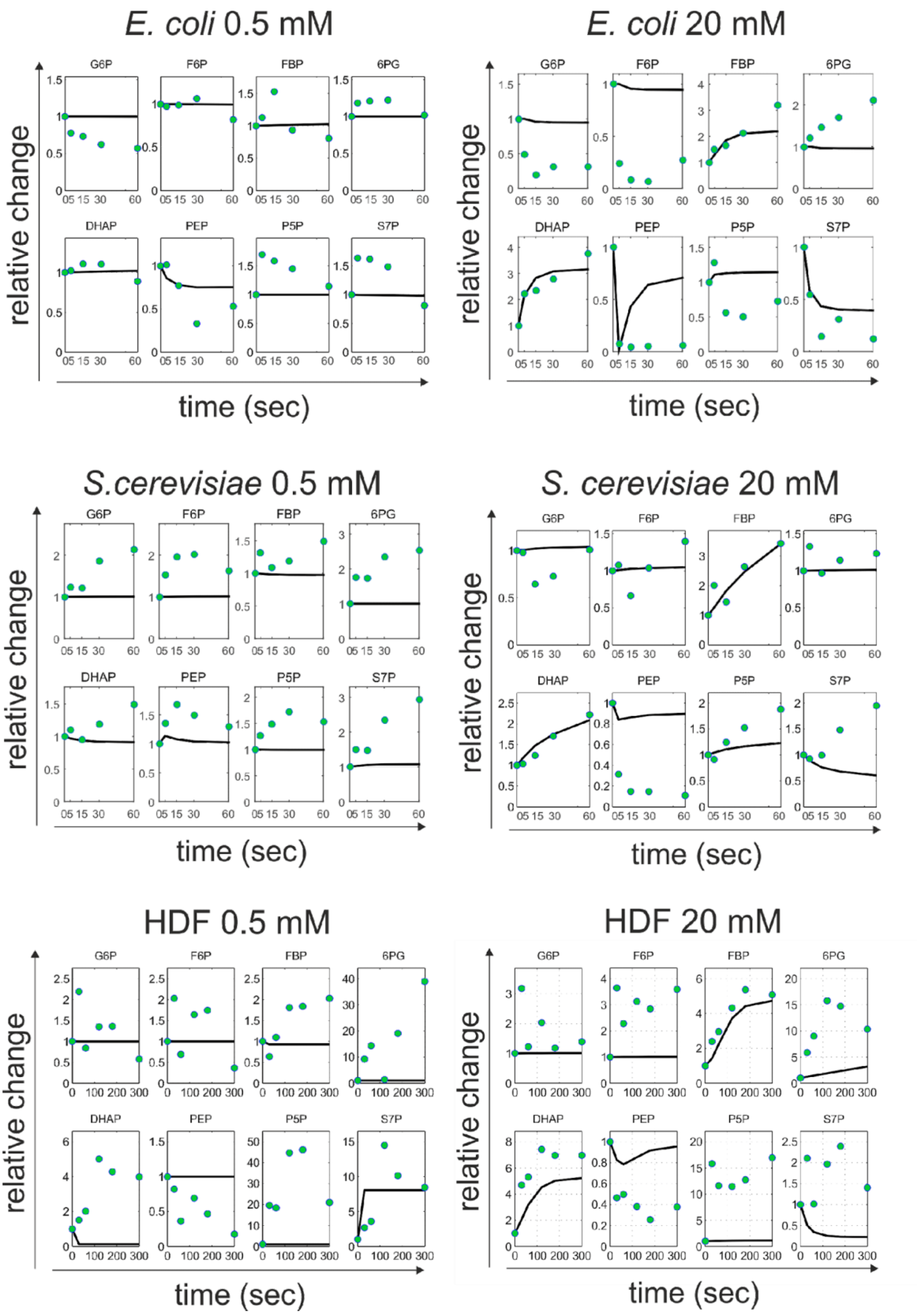

Figure S16: Base models (models amended with the ROS inhibition of GAP dehydrogenase) of *E. coli*, *S. cerevisiae* and human dermal fibroblasts (HDF) simulation results (black solid line) against the experimental data (green dots), in low (0.5 mM) and high (20 mM) concentrations of hydrogen peroxide stress. Y axis represents the relative change of a particular metabolite, compared to the untreated condition (time point 0). Related to Figure 5

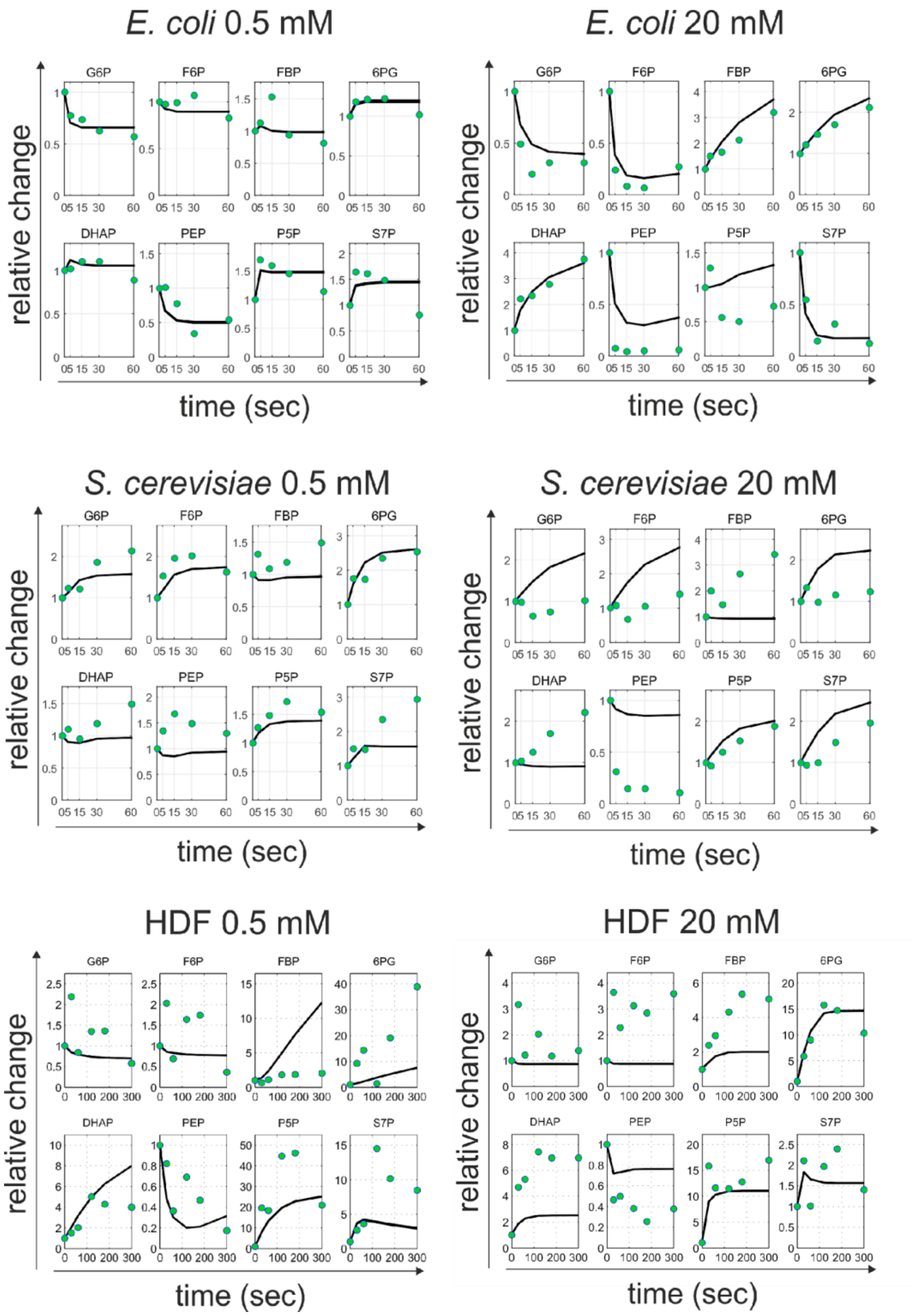

Figure S17: Simulation results (black solid lines) of the best performing models of E. coli, S. cerevisiae and human dermal fibroblasts (HDF) against the experimental data (green dots). Y-axis represents the relative change of a particular metabolite, compared to the untreated condition (time point 0). The best performing models include the following interactions (for the cases starting from upper left to bottom right): ***E. coli* 0.5 mM**: NADPH inhibition of G6P dehydrogenase and P5P activation of GND, ***E. coli* 20 mM**: NADPH inhibition of G6P dehydrogenase and PEP inhibition of PFK, ***S. cerevisiae* 0.5 mM**: NADPH inhibition of G6P dehydrogenase and PEP inhibition of G6P dehydrogenase, ***S. cerevisiae* 20 mM**: PYR inhibition of G6P dehydrogenase and NADPH activation of PFK, **HDF 0.5 mM**: NADPH inhibition of G6P dehydrogenase and NADPH inhibition of GND, **HDF 20 mM**: NADPH inhibition of G6P dehydrogenase and S7P activation of GND. Related to Figure 5.

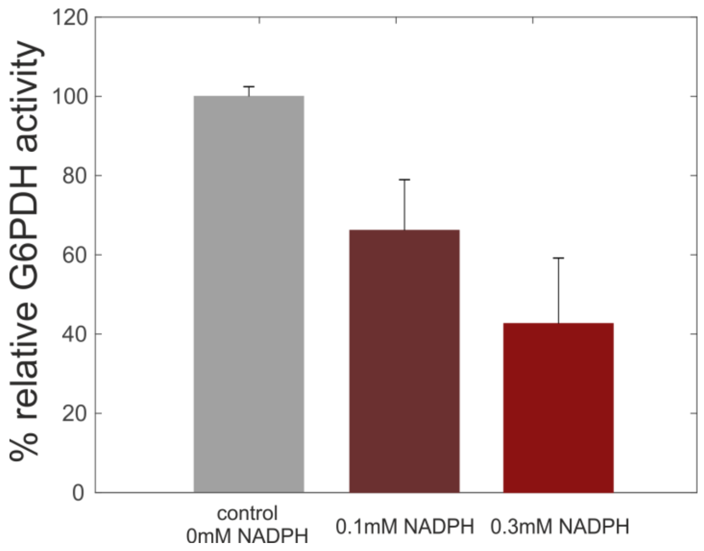

Figure S18: Percentage of relative G6P dehydrogenase activity in the presence of predicted inhibitor NADPH. Two concentrations were used: 0.1 mM and 0.3 mM of NADPH. Bars show the average activity and error bars denote the standard deviation, calculated from five individual replicates. Related to Figure 5.
